# Supplementary material for: Adaptive evolution shapes the present-day distribution of the thermal sensitivity of population growth rate
Source: PLoS Biol. 2020 Oct 16;18(10):e3000894. doi: 10.1371/journal.pbio.3000894 (PMC7592915; doi:10.1371/journal.pbio.3000894)
Supplement: S1 Appendix — (PDF) [file pbio.3000894.s001.pdf]

Adaptive evolution shapes the present-day distribution of the thermal sensitivity of population growth rate

Dimitrios - Georgios Kontopoulos[1,2*], Thomas P. Smith[2], Timothy G. Barraclough[2,3], and Samraat Pawar[2]

S1 Appendix

Contents

A Phylogeny reconstruction 2

B Dataset of thermal sensitivity estimates 3

C Phylogenetic comparative analyses 4

C.1 Analyses of the datasets of r_{max} TPCs . . . 4

C.2 Analyses of the dataset of phytoplankton TPCs after excluding Cyanobacteria . . . 9

C.3 Analyses of the net photosynthesis rate and respiration rate TPC datasets . . . 10

D Investigation of latitudinal associations for measures of thermal sensitivity 11

D.1 Latitudinal coverage of the dataset . . . 11

D.2 Fitted models using latitude as a continuous predictor . . . 11

D.3 Fitted models using binned latitude as predictor . . . 12

E Minimum generation times of microbes 14

F List of nucleotide sequences used for phylogeny reconstruction 14

1 Science and Solutions for a Changing Planet DTP, Imperial College London, London, United Kingdom.
2 Department of Life Sciences, Imperial College London, Silwood Park, Ascot, Berkshire, United Kingdom.
3 Department of Zoology, University of Oxford, Oxford, Oxfordshire, United Kingdom.
***** Corresponding author; e-mail: dgkontopoulos@gmail.com.

A Phylogeny reconstruction

The final tree produced by RAxML [1] and calibrated to relative time with DPPDiv [2] is shown in Fig A.

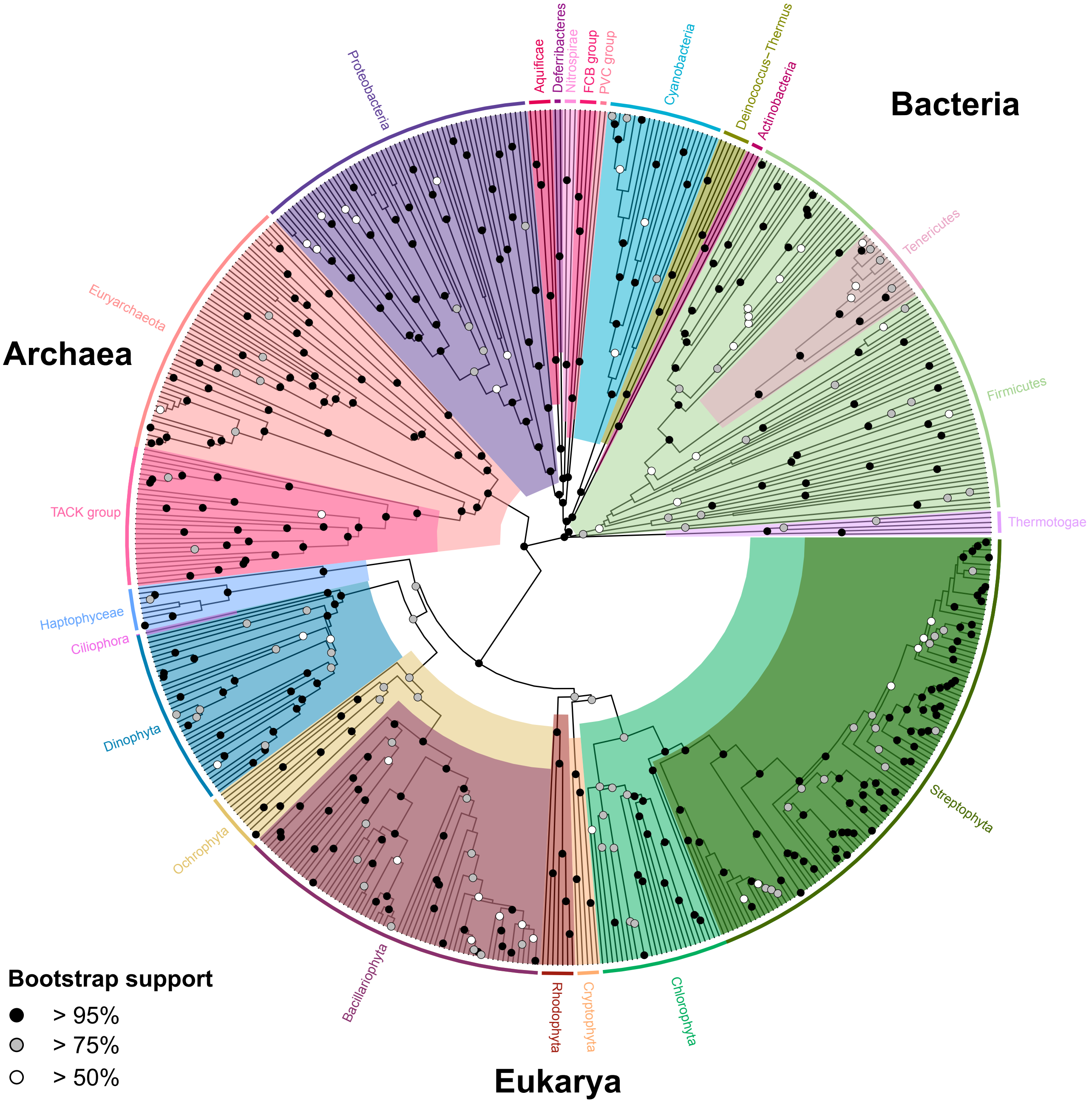

Fig A. The phylogeny generated in this study from which subtrees were extracted for comparative analyses. Colours indicate different phyla, whereas circles show the statistical support for each node, conditional to the topological constraints of the Open Tree of Life [3]. The phylogeny is available in NEXUS format at `https://doi.org/10.6084/m9.figshare.12816140.v1`.

B Dataset of thermal sensitivity estimates

The distributions of E and W_{op} values across the four datasets for species included in the phylogeny are shown in Fig B. Fig C shows the distributions of thermal sensitivity estimates of r_{max} across the six largest phyla of this study.

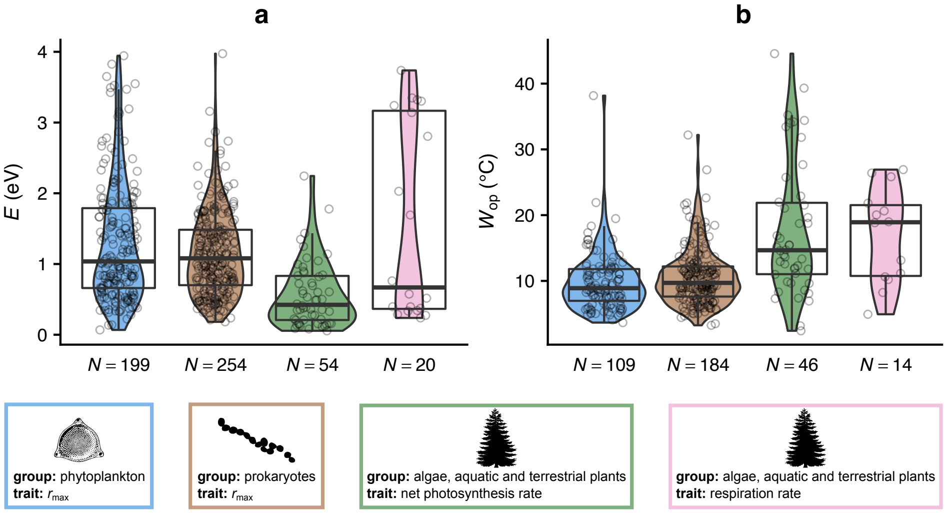

Fig B. All estimates of thermal sensitivity that were part of this study. Multiple estimates from the same species are not averaged but are included as separate data points. As experimentalists rarely measure trait performance across the entire TPC, for many TPCs it was not possible to robustly estimate both E and W_{op}. For this reason, E and W_{op} do not have the same sample size for each dataset. Overall, the distributions of thermal sensitivity parameters are not approximately Gaussian but asymmetric and not generally inflated at their boundaries. This indicates that the variation in thermal sensitivity is real and not purely due to measurement error. The raw data underlying this figure are available at `https://doi.org/10.6084/m9.figshare.12816140.v1`.

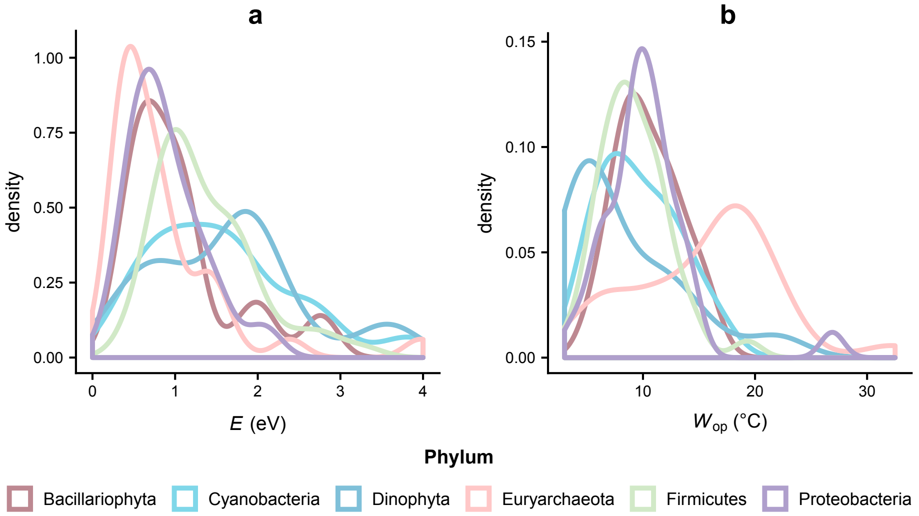

Fig C. The E and W_{op} distributions of the largest phyla in the study exhibit considerable overlap. Here, each species is represented by a single thermal sensitivity estimate. The increasing convergence of thermal sensitivity distributions even among evolutionarily remote phyla (e.g., between Cyanobacteria and Dinophyta or between Bacillariophyta and Proteobacteria) explains the intermediate phylogenetic heritability of thermal sensitivity. Moreover, it suggests that different values of E or W_{op} correspond to distinct thermal strategies which species can evolve largely regardless of their evolutionary background. The data underlying this figure are available at `https://doi.org/10.6084/m9.figshare.12816140.v1`.

C Phylogenetic comparative analyses

C.1 Analyses of the datasets of r_{max} TPCs

We first used MCMCglmm to estimate the phylogenetic heritabilities of our six TPC parameters by inferring their variance/covariance matrix, corrected for phylogeny. This allowed us to also extract the phenotypic correlation (r_{phe}) between E and W_{op} and, thus, to understand the relationship between the two thermal sensitivity measures (Fig D). Furthermore, the phenotypic correlation was broken down to its phylogenetically heritable component (r_{her}) and its residual component (r_{res}). The latter should be driven mostly by environmental effects. To understand why the relationship shown in Fig D arises, we numerically examined how W_{op} is affected by changes in E, and the sensitivity of their relationship to changes in B_0, T_{pk}, and E_D (Fig E).

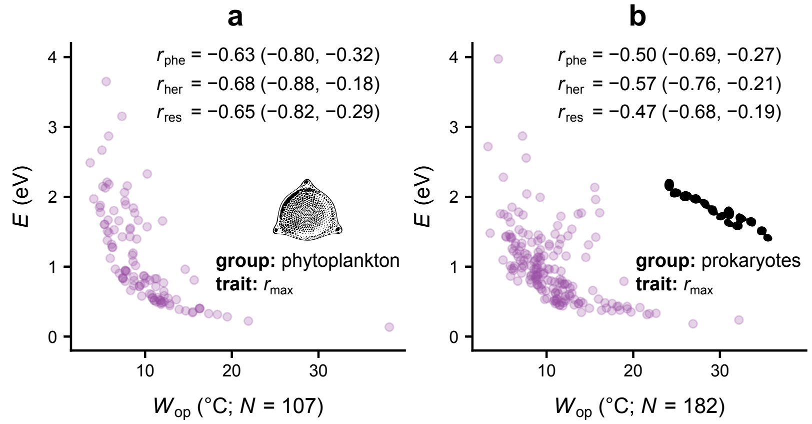

Fig D. Correlations between E and W_{op} among phytoplankton and prokaryotes, as estimated with MCMCglmm. Despite both being measures of thermal sensitivity, E and W_{op} are not perfectly correlated. The correlation coefficients shown are posterior distribution means, with values in parentheses indicating the 95% Highest Posterior Density interval. All correlation estimates were obtained for $\ln(E)$ and $\ln(W_{op})$, whereas here the parameters are shown untransformed. The data underlying this figure are available at `https://doi.org/10.6084/m9.figshare.12816140.v1`.

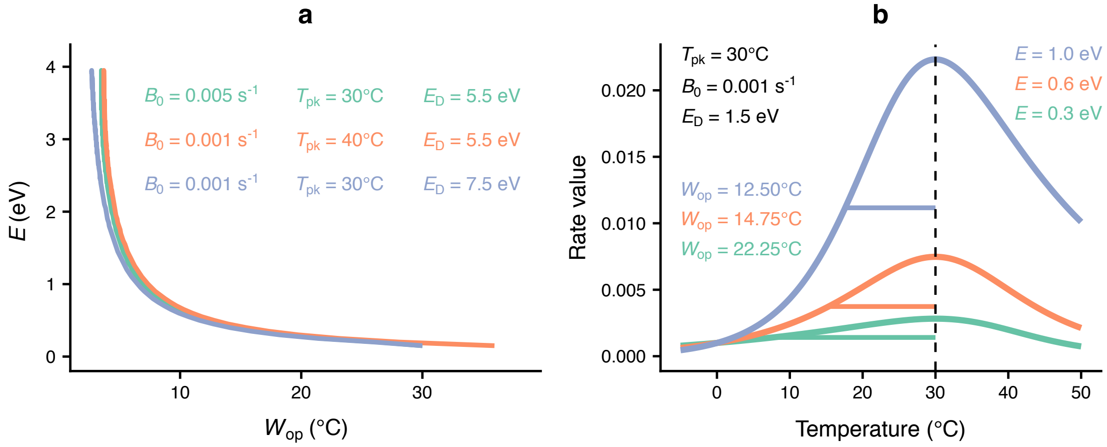

Fig E. The expected relationship of the operational niche width (W_{op}) and E in the Sharpe-Schoolfield model. (a) W_{op} always decreases with E, provided that the other parameters (B_0, T_{pk}, and E_D) do not vary substantially and systematically with E. This is illustrated here with three arbitrary fixed combinations of the other three parameters; the curves remain practically the same, irrespective of substantial variation in these other parameters. (b) An example illustrating how W_{op} always decreases with E when the other parameters are fixed (values shown in black). As E increases from 0.3 to 1 eV, W_{op} decreases from 22.25 to 12.50°C.

Besides MCMCglmm, we also estimated phylogenetic heritabilities using Rphylopars and BayesTraits and compared the resulting estimates with those of MCMCglmm (Fig F).

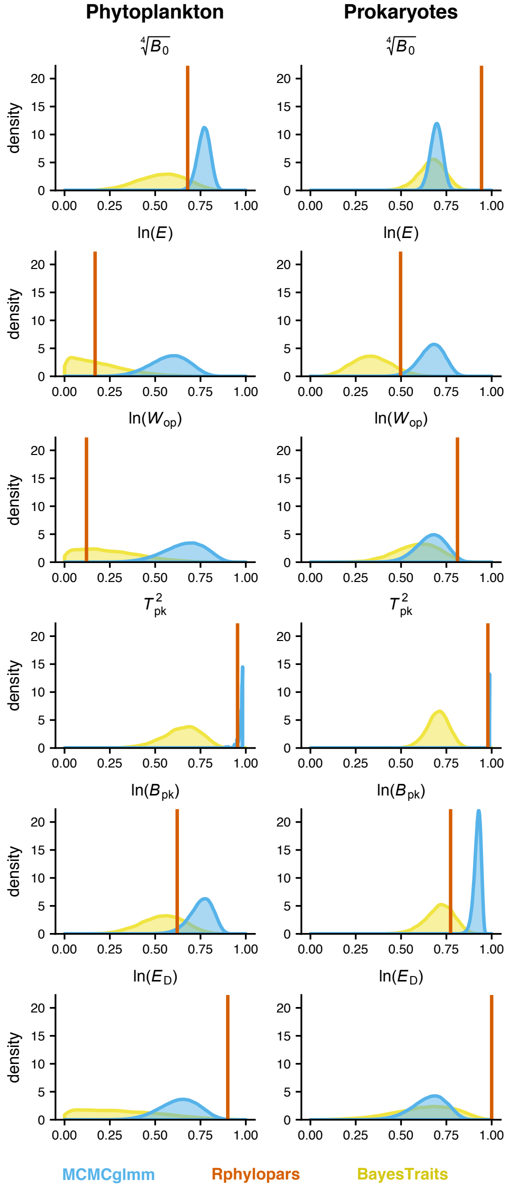

Fig F. Comparison of phylogenetic heritability estimates of MCMCglmm, Rphylopars, and BayesTraits. As MCMCglmm and BayesTraits estimate phylogenetic heritability using a Bayesian approach, the plots show their posterior distributions. Instead, point estimates are shown for Rphylopars. The phylogenetic heritability estimates obtained with Rphylopars are greater than zero for all TPC parameters. While the mean phylogenetic heritability estimates of BayesTraits are generally lower than those of MCMCglmm and Rphylopars, the lower bound of the 95% Highest Posterior Density interval of BayesTraits is always greater than zero (the lowest value is at $2 \cdot 10^{-5}$ for $\ln(E)$ among phytoplankton). Furthermore, the distributions of phylogenetic heritabilities of prokaryotes obtained with BayesTraits are much narrower and closer to those of MCMCglmm, compared to those obtained for phytoplankton with the two programs. This is consistent with an increase in the "signal-to-noise" ratio from phytoplankton to prokaryotes, given that the latter dataset is larger (Fig B). In any case, the observed differences in the estimates of MCMCglmm and BayesTraits may arise from i) differences in the priors used by the two methods, ii) accounting (or not) for the uncertainty of each TPC parameter estimate, or from iii) differences in the approaches employed for the estimation of missing TPC parameter values. The raw data underlying this figure are available at `https://doi.org/10.6084/m9.figshare.12816140.v1`.

To examine how the evolutionary rate of thermal sensitivity varies across the phylogeny, we fitted the stable model of trait evolution [4] (Fig 5 in the main text) but also the free model [5] (Fig G) and the Lévy model [6] (Fig H).

Finally, to better understand how species explore the parameter space of E, W_{op}, and T_{pk} (whose phylogenetic heritability is ≈ 1; see Figs 2 and F), we combined our two r_{max} datasets and divided the distributions of E, W_{op}, and T_{pk} into four discrete states (Fig I). Boundaries for these states were selected using the Jenks natural breaks clustering algorithm [7], as implemented in the BAMMtools R package (v. 2.1.6) [8]. To estimate the transition rates among states, we fitted the "all-rates-different" variant of the Mk model [9] with the `fitMk` function of the phytools R package (v. 0.6-60) [10].

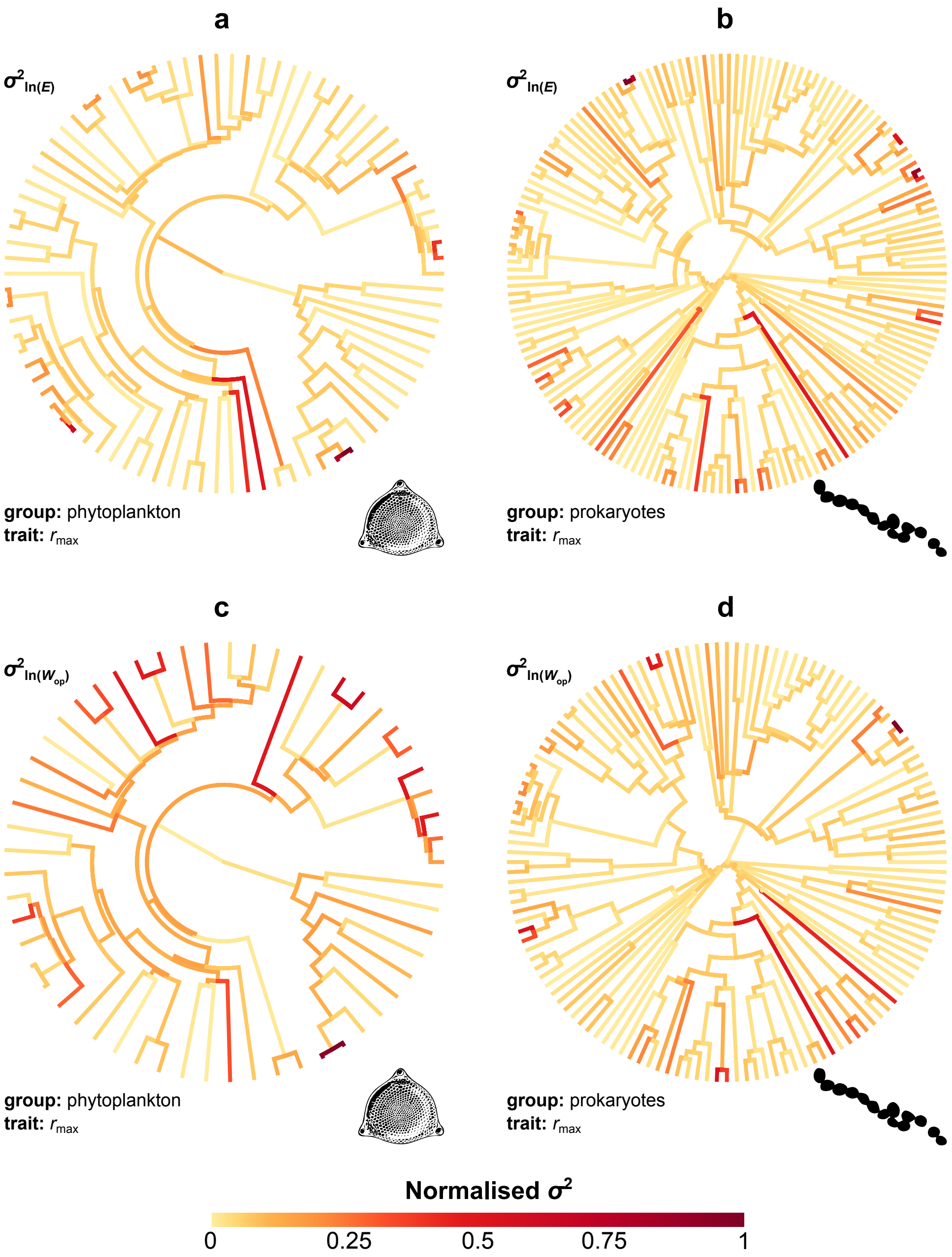

Fig G. Variation in the evolutionary rate across the phylogeny, as inferred with the free model. The results are qualitatively similar to those obtained with the stable model. The highest evolutionary rates (dark red and brown) generally appear in late-branching lineages across the phylogeny and are not clustered in specific clades. The raw data underlying this figure are available at `https://doi.org/10.6084/m9.figshare.12816140.v1`.

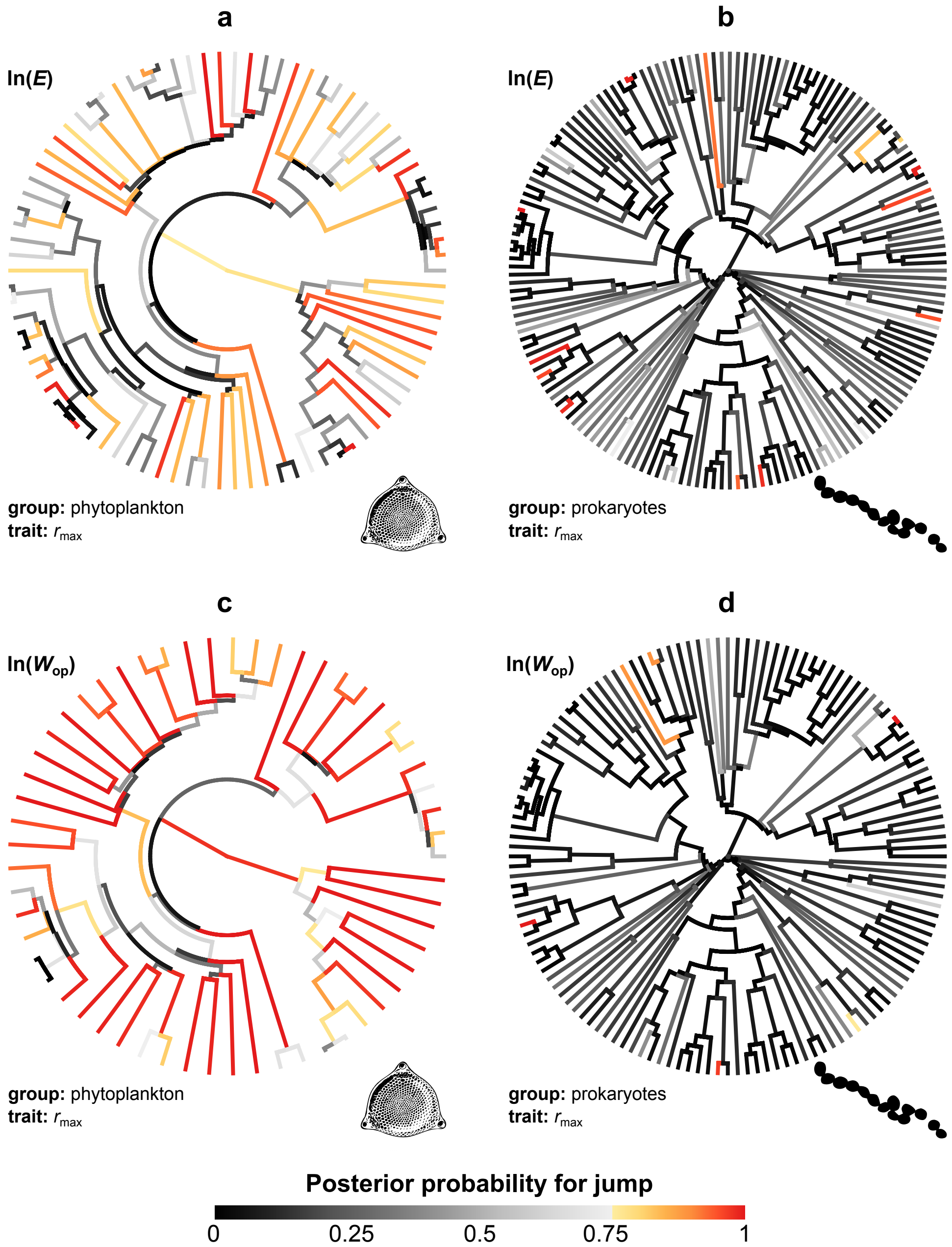

Fig H. Evolutionary jumps in thermal sensitivity across the phylogeny, as inferred with the Lévy model. The results are again qualitatively similar to those obtained with the stable model. Branches with a high posterior probability for the occurrence of a jump (shown in yellow to dark red) are distributed across the entire phylogeny and are not limited to specific clades. Note that in all cases, the Lévy model had a much lower AIC (between 20 and 147 units difference) than the constant-rate Brownian motion model (the null expectation). The raw data underlying this figure are available at `https://doi.org/10.6084/m9.figshare.12816140.v1`.

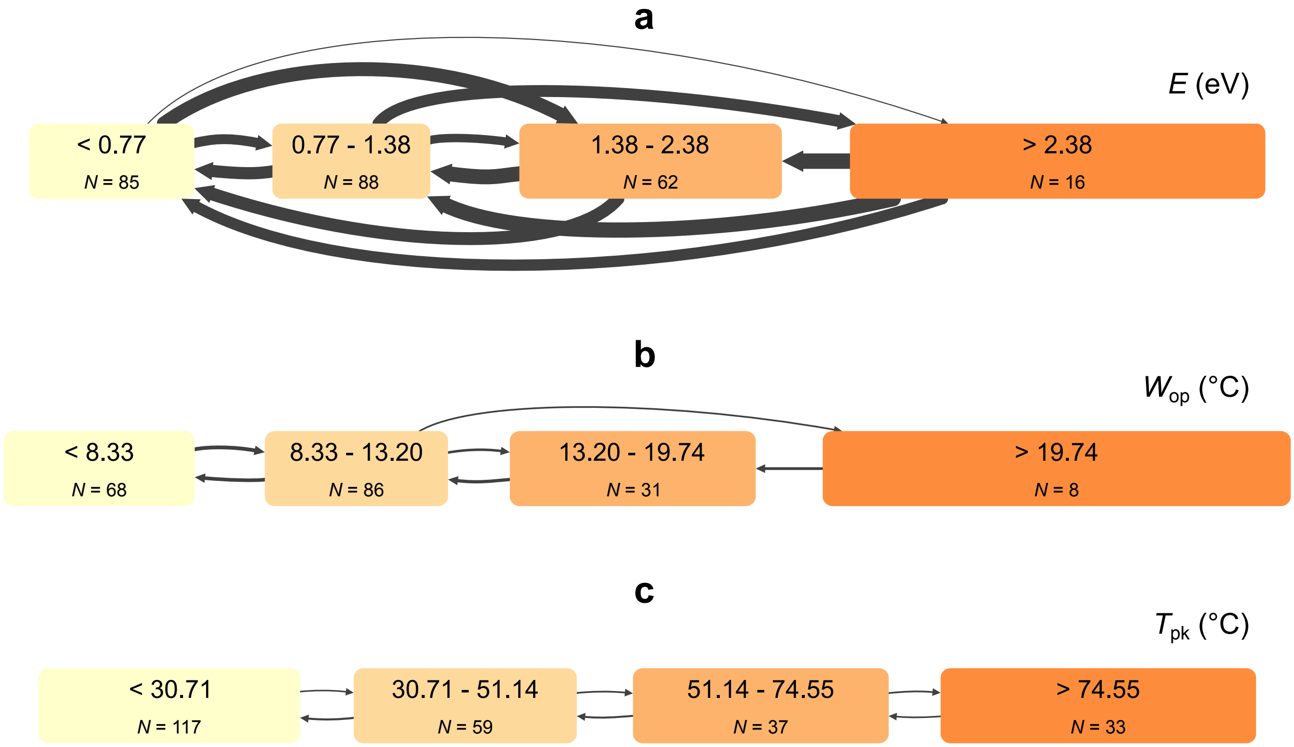

Fig I. Transitions in the discretized parameter space of E, $W_{\mathbf{op}}$, and $T_{\mathbf{pk}}$. The width of the edges represents the natural logarithm of the transition rate between states. Transitions between non-neighbouring states are very common for E (which captures the rise of the TPC), rare for W_{op} (which captures both the rise and the peak of the TPC), and never observed for T_{pk} (which captures the peak of the TPC). It is worth pointing out that T_{pk} also exhibits the lowest transition rates between neighbouring states among the three TPC parameters. These results are consistent with the phylogenetic heritability estimates shown in Fig 2 in the main text. The raw data underlying this figure are available at `https://doi.org/10.6084/m9.figshare.12816140.v1`.

C.2 Analyses of the dataset of phytoplankton TPCs after excluding Cyanobacteria

Removing Cyanobacteria from the phytoplankton dataset led to qualitatively identical results in our phylogenetic analyses (Figs J and K).

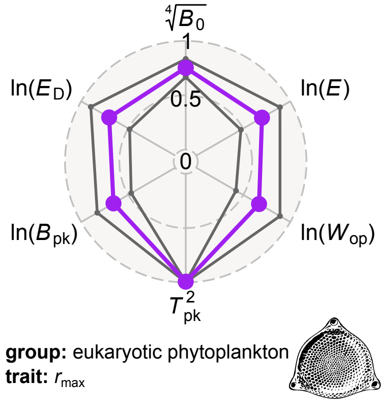

Fig J. Phylogenetic heritabilities of TPC parameters of eukaryotic phytoplankton. The main differences between these results and those using the entire phytoplankton dataset (Fig 2A in the main text) were that, here, ln(E) and ln(B_{pk}) have slightly higher/lower phylogenetic heritabilities respectively. The former is expected as the thermal sensitivity distribution of Cyanobacteria is very similar to that of Dinophyta (Fig 4 in the main text), despite the long evolutionary distance between them. Therefore, the exclusion of Cyanobacteria would necessarily increase the phylogenetic heritability of thermal sensitivity. Similarly, ln(B_{pk}) in prokaryotes is more phylogenetically heritable than in phytoplankton (Fig 2 in the main text), explaining the further decrease in its phylogenetic heritability when Cyanobacteria are excluded. The data underlying this figure are available at `https://doi.org/10.6084/m9.figshare.12816140.v1`.

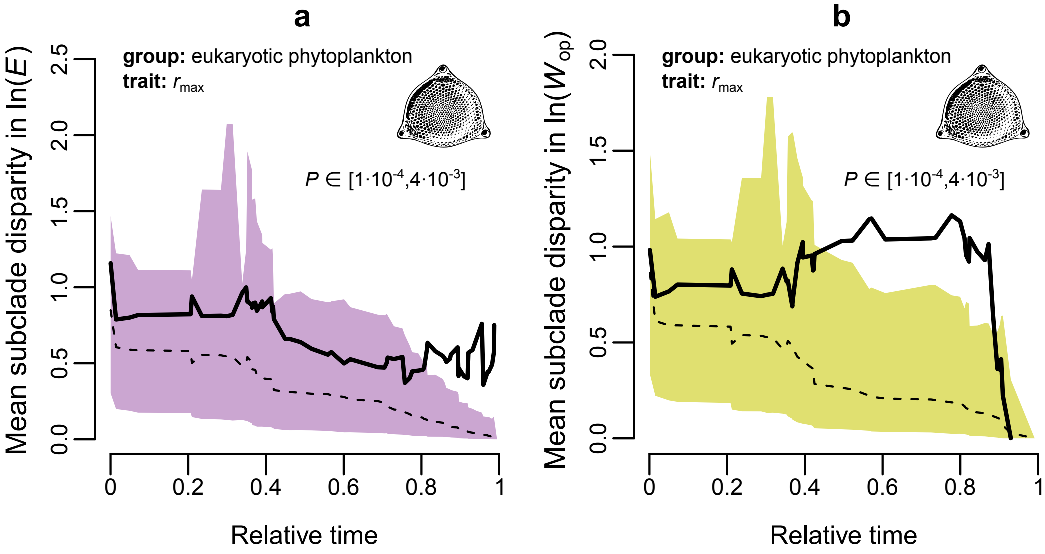

Fig K. The mean subclade disparity in thermal sensitivity tends to increase with time across phytoplankton, even after excluding Cyanobacteria. The pattern is slightly weaker (albeit still present) due to the lower sample size. The raw data underlying this figure are available at `https://doi.org/10.6084/m9.figshare.12816140.v1`.

C.3 Analyses of the net photosynthesis rate and respiration rate TPC datasets

The visualization of the evolution of thermal sensitivity of net photosynthesis rate and respiration rate revealed similar patterns to those of the thermal sensitivity of $r_{\max}$ (Fig 6 in the main text). Thermal sensitivity values do not evolve gradually and tightly around a central value ($\widehat{\theta}$), but explore large parts of the parameter space due to bursts of rapid evolution.

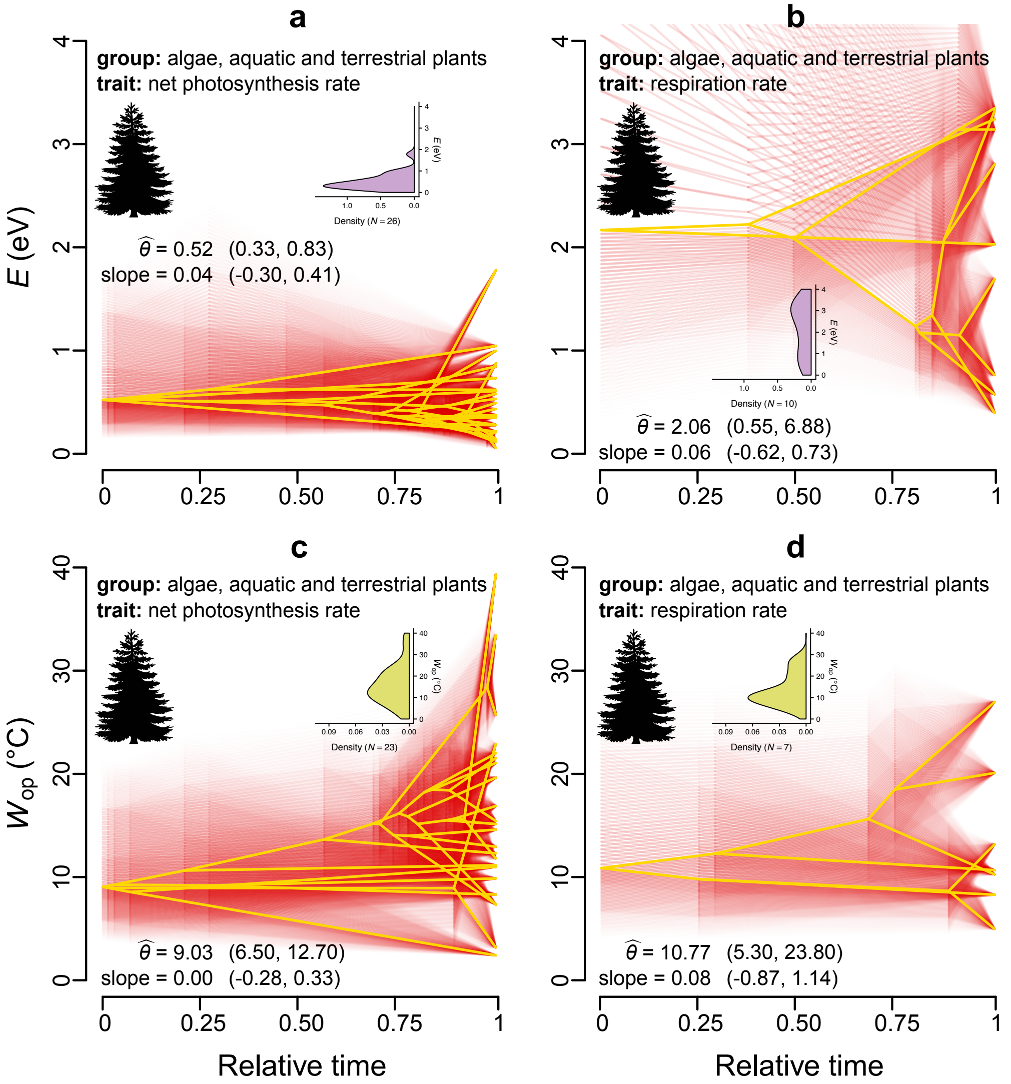

Fig L. Evolution of the thermal sensitivities of net photosynthesis rate and respiration rate through time. The inset figures show the density distributions of E and W_{op} values of extant species in the dataset. The raw data underlying this figure are available at `https://doi.org/10.6084/m9.figshare.12816140.v1`.

D Investigation of latitudinal associations for measures of thermal sensitivity

D.1 Latitudinal coverage of the dataset

The latitudes of species/strains from which we obtained estimates of thermal sensitivity (E and W_{op}) are shown in Fig M.

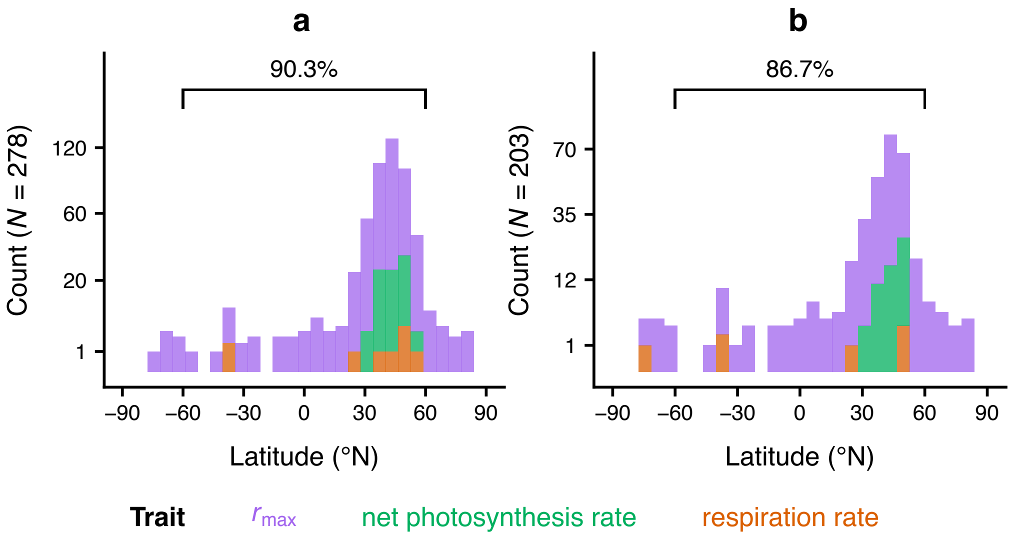

Fig M. Latitudinal distribution of E (a) and W_{op} (b) estimates in this study. Most estimates are from species/strains found at low and intermediate latitudes. The percentage at the top of each panel indicates the fraction of estimates from latitudes between -60°N and 60°N. Note that values along the vertical axes do not increase linearly. The raw data underlying this figure are available at `https://doi.org/10.6084/m9.figshare.12816140.v1`.

D.2 Fitted models using latitude as a continuous predictor

We rejected models that had one or more non-intercept coefficients with a 95% HPD interval that included zero. We then used DIC to identify the most appropriate model among those remaining.

Model	Phylogenetic correction	Mean DIC
$\ln(E) \sim$ Intercept + cos(Latitude) + Trait identity	✓	535.2840
$\ln(E) \sim$ Intercept + |Latitude| + Trait identity	✓	536.0311
$\ln(E) \sim$ Intercept + Trait identity	✓	538.7041
$\ln(E) \sim$ Intercept	✓	569.9043
$\ln(E) \sim$ Intercept + cos(Latitude) + Trait identity	✗	489.5730
$\ln(E) \sim$ Intercept + |Latitude| + Trait identity	✗	488.8540
$\ln(E) \sim$ Intercept + cos(Latitude)	✗	511.4035
$\ln(E) \sim$ Intercept + |Latitude|	✗	510.1707
$\ln(E) \sim$ Intercept + Trait identity	✗	492.4570
$\ln(E) \sim$ Intercept	✗	514.0522

Table A. Candidate models with ln(E) as the response variable.

Model	Phylogenetic correction	Mean DIC
$\ln(W_{\text{op}}) \sim$ Intercept	✓	148.6599
$\ln(W_{\text{op}}) \sim$ Intercept + Trait identity	✗	144.8427
$\ln(W_{\text{op}}) \sim$ Intercept	✗	147.4493

Table B. Candidate models with ln(W_{op}) as the response variable.

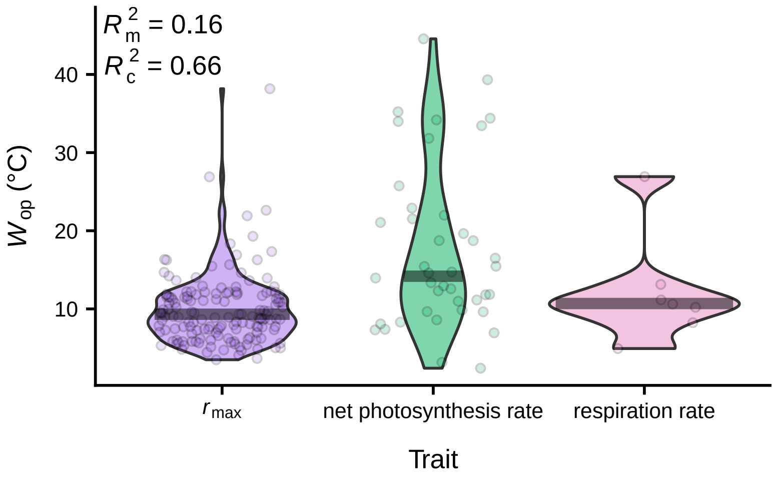

Fig N. The most appropriate model for ln(W_{op}) does not include latitude as a predictor variable. Trait identity on its own accounts for 16% of variance, whereas also incorporating species identity (as a random effect on the intercept) results in 66% of the variance being explained. Grey horizontal lines denote the inferred intercept for each trait. The data underlying this figure are available at `https://doi.org/10.6084/m9.figshare.12816140.v1`.

D.3 Fitted models using binned latitude as predictor

Model	Phylogenetic correction	Mean DIC
$\ln(E) \sim$ Intercept + $\lvert\text{Latitude}\rvert_{0-30}$ + $\lvert\text{Latitude}\rvert_{>60}$ + Trait identity	✓	535.8695
$\ln(E) \sim$ Intercept + $\lvert\text{Latitude}\rvert_{0-30}$ + $\lvert\text{Latitude}\rvert_{>60}$	✓	565.1168
$\ln(E) \sim$ Intercept + Trait identity	✓	538.7041
$\ln(E) \sim$ Intercept	✓	569.9043
$\ln(E) \sim$ Intercept + $\lvert\text{Latitude}\rvert_{0-30}$ + $\lvert\text{Latitude}\rvert_{>60}$ + Trait identity	✗	485.0223
$\ln(E) \sim$ Intercept + $\lvert\text{Latitude}\rvert_{0-30}$ + $\lvert\text{Latitude}\rvert_{>60}$	✗	504.8906
$\ln(E) \sim$ Intercept + Trait identity	✗	492.4570
$\ln(E) \sim$ Intercept	✗	514.0522

Table C. Candidate models for the effects of binned absolute latitude on ln(E).

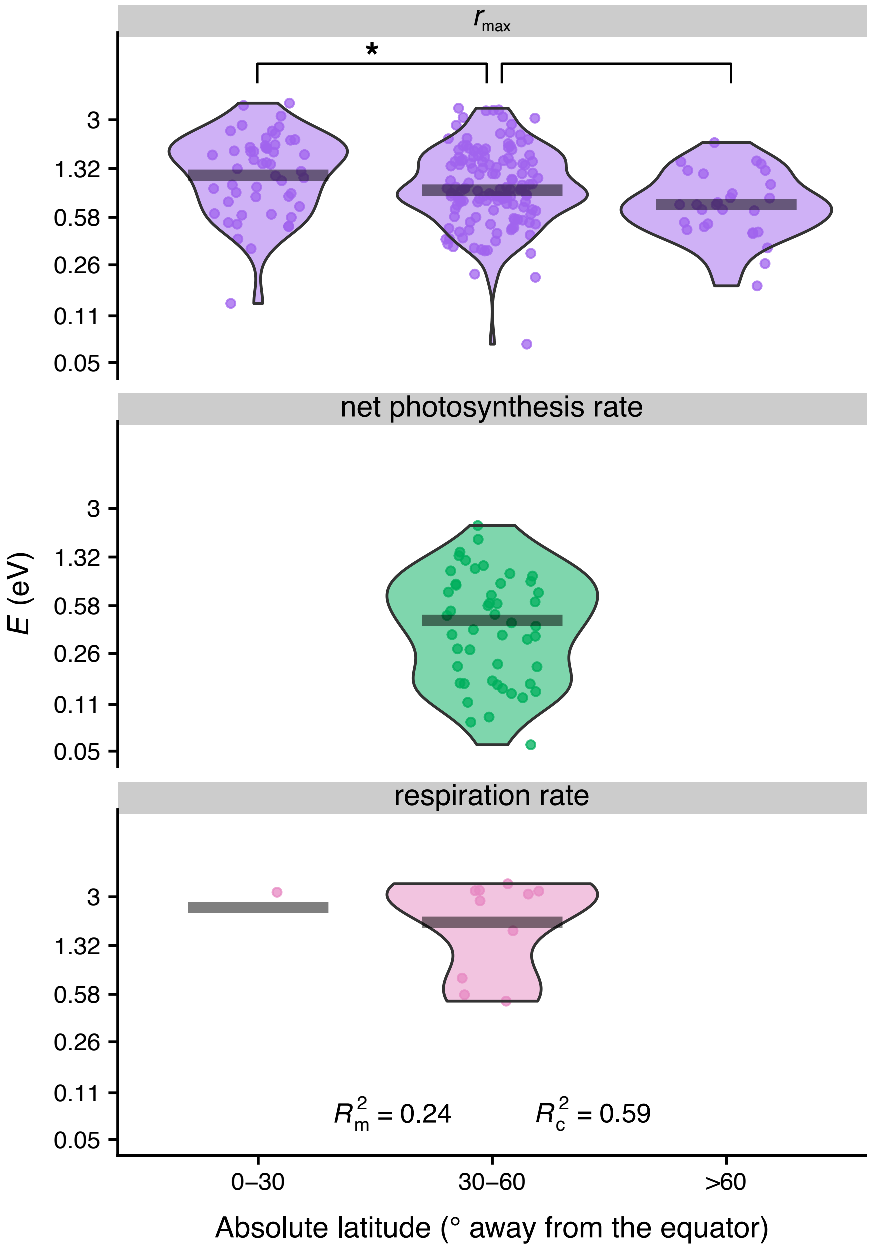

Fig O. Distribution of E estimates across low, intermediate, and high latitudes. Species/strains found in low latitudes tend to have slightly higher E values than those in intermediate latitudes. In contrast, the E distributions of intermediate and high latitudes were statistically indistinguishable. The data underlying this figure are available at `https://doi.org/10.6084/m9.figshare.12816140.v1`.

E Minimum generation times of microbes

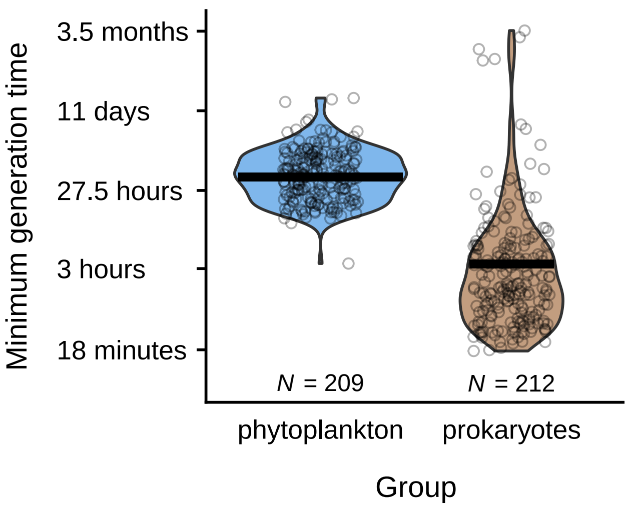

Fig P. Distribution of minimum generation time estimates for phytoplankton and prokaryotes. Data points were obtained by taking the inverse of all B_{pk} estimates. Horizontal bars represent the phylogenetically-corrected median values, i.e., the inverse of the intercept of B_{pk} from the multi-response regression models that we fitted with MCMCglmm (see the "Estimation of phylogenetic heritability for all TPC parameters using MCMCglmm, Rphylopars, and BayesTraits" subsection of the Methods in the main text). The data underlying this figure are available at `https://doi.org/10.6084/m9.figshare.12816140.v1`.

F List of nucleotide sequences used for phylogeny reconstruction

Table D. Species names and Accession IDs of small subunit rRNA gene sequences that were used in this study.

**Species**	**Accession ID**
Abies alba	GenBank: DQ371809.1
Abutilon theophrasti	GenBank: DQ287985.1
Acer rubrum	GenBank: U42494.1
Acetobacterium bakii	NCBI Reference Sequence: NR_026329.1
Acetobacterium carbinolicum	NCBI Reference Sequence: NR_026325.1
Acetobacterium fimetarium	NCBI Reference Sequence: NR_026328.1
Acetobacterium paludosum	NCBI Reference Sequence: NR_026327.1
Acetobacterium tundrae	NCBI Reference Sequence: NR_028934.1
Acetogenium kivui	NCBI Reference Sequence: NR_044617.1
Acidianus brierleyi	NCBI Reference Sequence: NR_043409.1
Acidianus infernus	NCBI Reference Sequence: NR_043431.1
Acidianus manzaensis	GenBank: EU563854.1
Acidibacter ferrireducens	NCBI Reference Sequence: NR_126260.1
Acidicaldus organivorus	NCBI Reference Sequence: NR_042752.1
Acidilobus aceticus	NCBI Reference Sequence: NR_041774.1
Acidilobus sulfurireducens	NCBI Reference Sequence: NR_115940.1
Acidimicrobium ferrooxidans	NCBI Reference Sequence: NR_074390.1
Acidithiobacillus caldus	GenBank: KJ944319.1
Acidithiobacillus ferrivorans	GenBank: KJ679874.1
Acidithiobacillus ferrooxidans	GenBank: DQ062118.1
Acidithiobacillus thiooxidans	GenBank: DQ834372.1
Acidocella aromatica	GenBank: AF253413.1

Table D – *Continued from previous page*

**Species**	**Accession ID**
Aeribacillus pallidus	NCBI Reference Sequence: NR_026515.1
Aeromonas hydrophila	GenBank: M59148.1
Aeropyrum pernix	NCBI Reference Sequence: NR_043417.1
Aldrovanda vesiculosa	GenBank: AY096114.1
Alexandrium catenella	GenBank: AJ535392.1
Alexandrium fundyense	GenBank: KF908796.1
Alexandrium minutum	GenBank: U27499.1
Alexandrium monilatum	GenBank: AY883005.1
Alexandrium ostenfeldii	GenBank: U27500.1
Alexandrium tamarense	GenBank: AJ415510.1
Alkaliphilus transvaalensis	GenBank: AJ630291.1
Amphidinium klebsii	GenBank: EU046335.1
Amphiprora paludosa	GenBank: AY485468.1
Anabaena bergii	GenBank: AF160256.1
Anabaena macrospora	GenBank: AJ293115.1
Anabaena spiroides	GenBank: AB271212.1
Anabaena ucrainica	GenBank: AB551452.1
Anabaena variabilis	GenBank: AB016520.1
Ankistrodesmus falcatus var. tumidus	GenBank: JQ315498.1
Antirrhinum majus	GenBank: AJ236047.1
Aphanizomenon flosaquae	GenBank: HE975013.1
Aphanizomenon gracile	GenBank: AJ293127.1
Aphanizomenon ovalisporum	GenBank: FM177484.1
Aplectrum hyemale	GenBank: U59937.1
Arabidopsis thaliana	NCBI Reference Sequence: NR_141642.1
Arbutus unedo	GenBank: AF206853.1
Archaeoglobus veneficus	NCBI Reference Sequence: NR_102885.1
Aristotelia serrata	GenBank: GU476422.1
Asterionella formosa	GenBank: AM712617.1
Asterionellopsis glacialis	GenBank: X77701.1
Aulacoseira baicalensis	GenBank: AY121821.1
Aulacoseira granulata	GenBank: AB430586.1
Bacillus acidocaldarius	GenBank: X60742.1
Bacillus caldotenax	GenBank: AY608937.1
Bacillus cereus	GenBank: KU198623.1
Bacillus infernus	NCBI Reference Sequence: NR_027227.1
Bacillus megaterium	GenBank: HM371417.1
Bacillus subtilis	GenBank: AY728013.1
Beta vulgaris	GenBank: FJ669720.1
Betula papyrifera	GenBank: L00971.1
Betula pendula	GenBank: GU476453.1
Brassica oleracea	GenBank: KT225359.1
Brassica rapa	GenBank: LC009534.1
Brochothrix thermosphacta	GenBank: M58798.1
Bryum argenteum	GenBank: U18529.1
Caldicellulosiruptor obsidiansis	NCBI Reference Sequence: NR_117295.1
Caldisphaera draconis	NCBI Reference Sequence: NR_115941.1
Caldivirga maquilingensis	NCBI Reference Sequence: NG_042069.1
Caloramator indicus	NCBI Reference Sequence: NR_026134.1
Caloranaerobacter azorensis	NCBI Reference Sequence: NR_028919.1
Candidatus Brocadia sinica	GenBank: KT023578.1
Capsella bursa-pastoris	GenBank: KT459181.1

Table D – *Continued from previous page*

**Species**	**Accession ID**
Capsicum annuum	GenBank: EF564281.1
Carya glabra	GenBank: AF206880.1
Caulerpa serrulata	GenBank: JQ745683.1
Ceratium furca	GenBank: AJ276699.1
Ceratium fusus	GenBank: AF022153.1
Ceratophyllum demersum	GenBank: U42517.1
Chaetoceros debilis	GenBank: AB847419.1
Chamaebatiaria millefolium	GenBank: DQ886366.1
Chamerion angustifolium	GenBank: AH001636.2
Chatonella marina	GenBank: AB217627.1
Chenopodium album	GenBank: HQ827790.1
Chlamydomonas reinhardtii	GenBank: KF864473.1
Chlamydomonas subcaudata	GenBank: AJ781310.1
Chlorella ellipsoidea	GenBank: X63520.1
Chlorella pyrenoidosa	GenBank: AB240151.1
Chlorella saccharophila	GenBank: AB183577.1
Chlorella sorokiniana	GenBank: EU402596.1
Chlorella vulgaris	GenBank: HQ702325.1
Chlorobium tepidum	NCBI Reference Sequence: NR_044685.2
Chondrus crispus	GenBank: DQ317002.1
Chroomonas salina	GenBank: GU983864.1
Chrysanthemum morifolium	GenBank: KJ870235.1
Cicer arietinum	GenBank: AJ011011.4
Citrus aurantium	GenBank: U38312.1
Citrus limon	GenBank: KJ740202.1
Cladophora glomerata	GenBank: AB665579.1
Closterium acerosum	GenBank: AF352230.1
Clostridium autoethanogenum	NCBI Reference Sequence: NR_119283.1
Clostridium fervidus	GenBank: L09187.1
Clostridium paradoxum	NCBI Reference Sequence: NR_119327.1
Clostridium perfringens	GenBank: LC037206.1
Clostridium thermoalcaliphilum	GenBank: FR749953.1
Clostridium thermohydrosulfuricum	NCBI Reference Sequence: NR_044618.1
Clostridium thermosuccinogenes	GenBank: Y18180.1
Clostridium thermosulfurogenes	GenBank: HG324062.2
Coccolithus pelagicus	GenBank: AJ246261.1
Cochlodinium polykrikoides	GenBank: EU418971.1
Coelastrum microporum	GenBank: JQ315527.1
Colwellia demingiae	NCBI Reference Sequence: NR_118860.1
Colwellia hornerae	NCBI Reference Sequence: NR_118861.1
Colwellia psychrerythraea	NCBI Reference Sequence: NR_037047.1
Colwellia psychrotropica	NCBI Reference Sequence: NR_026055.1
Coolia monotis	GenBank: EF492487.1
Coscinodiscus concinnus	GenBank: HQ912681.1
Coscinodiscus granii	GenBank: AY485495.1
Coscinodiscus jonesianus	GenBank: KJ577852.1
Cosmarium biretum	GenBank: AM920339.1
Cosmarium botrytis	GenBank: AM920378.1
Cosmarium crenatum	GenBank: AM920370.1
Cosmarium meneghinii	GenBank: AM920366.1
Cosmarium punctulatum	GenBank: AM920373.1
Cosmarium subprotumidum	GenBank: AM920375.1

Table D – *Continued from previous page*

Species	Accession ID
Cryptomonas erosa	GenBank: AM396361.1
Cryptomonas marssonii	GenBank: EU163586.1
Cryptomonas ovata	GenBank: KC928318.1
Cucumis sativus	GenBank: AF206894.1
Cyclotella cryptica	GenBank: AY485499.1
Cyclotella meneghiniana	GenBank: HM805030.1
Cylindrospermopsis raciborskii	GenBank: AF516730.1
Cylindrotheca closterium	GenBank: GQ468542.1
Cymodocea nodosa	GenBank: KT200607.1
Daucus carota	GenBank: GQ380561.1
Deferribacter thermophilus	NCBI Reference Sequence: NR_026043.1
Deinococcus geothermalis	NCBI Reference Sequence: NR_074342.1
Deinococcus murrayi	NCBI Reference Sequence: NR_026416.1
Desmarestia anceps	GenBank: HE866895.1
Desmidium swartzii	GenBank: AJ428133.1
Desulfitobacterium dehalogenans	GenBank: L28946.1
Desulfobacter curvatus	NCBI Reference Sequence: NR_041851.1
Desulfofaba gelida	NCBI Reference Sequence: NR_028730.1
Desulfofrigus fragile	NCBI Reference Sequence: NR_028732.1
Desulfofrigus oceanense	NCBI Reference Sequence: NR_028731.1
Desulforhopalus vacuolatus	NCBI Reference Sequence: NR_044653.1
Desulfotalea arctica	NCBI Reference Sequence: NR_024949.1
Desulfotalea psychrophila	NCBI Reference Sequence: NR_028729.1
Desulfotomaculum alkaliphilum	NCBI Reference Sequence: NR_024947.1
Desulfotomaculum putei	GenBank: HM228397.1
Desulfovibrio desulfuricans	NCBI Reference Sequence: NR_036778.1
Desulfovibrio profundus	NCBI Reference Sequence: NR_114641.1
Desulfovibrio salexigens	NCBI Reference Sequence: NR_102801.1
Desulfurobacterium crinifex	NCBI Reference Sequence: NR_114880.1
Desulfuromonas michiganensis	GenBank: AF357915.2
Detonula confervacea	GenBank: HQ912617.1
Diapensia lapponica	GenBank: AF419794.1
Dinobryon divergens	GenBank: EU025020.1
Ditylum brightwellii	GenBank: X85386.2
Dunaliella tertiolecta	GenBank: EF473747.1
Egeria densa	GenBank: JF975484.1
Elodea canadensis	GenBank: AF168841.1
Emiliania huxleyi	GenBank: KC404141.1
Enterococcus faecalis	GenBank: EU887827.1
Enteromorpha intestinalis	GenBank: AJ000040.1
Erwinia amylovora	GenBank: KM597069.1
Escherichia coli	GenBank: AB269763.1
Eucalyptus globulus	GenBank: HQ456544.1
Eucampia zodiacus	GenBank: KC309495.1
Eucheuma isiforme	GenBank: U25438.1
Ferroglobus placidus	NCBI Reference Sequence: NR_074531.1
Ferroplasma acidarmanus	GenBank: AF145441.1
Ferroplasma acidiphilum	GenBank: AF513710.1
Ferroplasma cupricumulans	GenBank: AY907888.1
Fervidobacterium pennavorans	GenBank: EF565822.1
Fibrocapsa japonica	GenBank: AY788931.1
Flavobacterium limicola	GenBank: AB075232.1

Table D – *Continued from previous page*

Species	Accession ID
Flexistipes sinusarabici	NCBI Reference Sequence: NR_074881.1
Fontinalis antipyretica	GenBank: AF023714.1
Fragilaria barbararum	GenBank: AJ971376.1
Fragilaria crotonensis	GenBank: AM712616.1
Fragilariopsis cylindrus	GenBank: EF140624.1
Fragilariopsis kerguelensis	GenBank: KJ866919.1
Fucus gardneri	GenBank: HQ710578.1
Gambierdiscus toxicus	GenBank: EF202890.1
Gelidibacter gilvus	NCBI Reference Sequence: NR_041693.1
Geobacillus caldoxylosilyticus	GenBank: AY608951.1
Geobacillus stearothermophilus	GenBank: EF025325.1
Geobacillus thermodenitrificans	GenBank: AJ785764.1
Geobacillus thermoleovorans	GenBank: JQ343209.1
Geoglobus ahangari	NCBI Reference Sequence: NR_041788.1
Gephyrocapsa oceanica	GenBank: KC404159.1
Gerbera jamesonii	GenBank: AF107576.1
Glaciecola punicea	NCBI Reference Sequence: NR_036866.1
Glycine max	GenBank: X02623.1
Gonatozygon monotaenium	GenBank: AJ428084.1
Gossypium hirsutum	GenBank: L24145.1
Gracilaria verrucosa	GenBank: M33638.1
Grammonema striatula	GenBank: X77704.1
Guinardia flaccida	GenBank: AJ535191.1
Gymnodinium breve	GenBank: AF172714.1
Gymnodinium catenatum	GenBank: AF022193.1
Gymnodinium mikimotoi	GenBank: AF022195.1
Gymnodinium sanguineum	GenBank: AJ415513.1
Gymnodinium veneficum	GenBank: AF172712.1
Gyrodinium aureolum	GenBank: AF172713.1
Gyrodinium instriatum	GenBank: DQ084522.1
Haematococcus pluvialis	GenBank: JQ315539.1
Haloanaerobium alcaliphilum	GenBank: KU180221.1
Haloanaerobium lacusroseus	NCBI Reference Sequence: NR_025924.1
Haloarcula vallismortis	NCBI Reference Sequence: NR_116083.1
Halobacterium salinarum	GenBank: AB663362.1
Halobaculum gomorrense	GenBank: L37444.1
Halococcus morrhuae	NCBI Reference Sequence: NR_043387.1
Haloferax volcanii	NCBI Reference Sequence: NR_113448.1
Halogeometricum borinquense	NCBI Reference Sequence: NR_028170.1
Halomonas campisalis	GenBank: DQ077908.1
Halomonas elongata	GenBank: KU053958.1
Halomonas marina	GenBank: AJ306890.1
Halomonas subglaciescola	NCBI Reference Sequence: NR_042067.1
Halonatronum saccharophilum	NCBI Reference Sequence: NR_042717.1
Halorubrum saccharovorum	NCBI Reference Sequence: NR_113484.1
Haloterrigena turkmenica	NCBI Reference Sequence: NR_113515.1
Helianthus annuus	GenBank: AF107577.1
Heliobacillus mobilis	NCBI Reference Sequence: NR_040957.1
Heliobacterium modesticaldum	NCBI Reference Sequence: NR_074517.1
Heterocapsa circularisquama	GenBank: LC054932.1
Heterocapsa triquetra	GenBank: AF022198.1
Heterosigma akashiwo	GenBank: AB217869.1

Table D – *Continued from previous page*

**Species**	**Accession ID**
Hordeum vulgare	GenBank: AH001585.2
Hormidium flaccidum	GenBank: M95613.1
Hydrilla verticillata	GenBank: KM982363.1
Hydrogenophaga pseudoflava	NCBI Reference Sequence: NR_028717.1
Hydrogenophilus hirschii	NCBI Reference Sequence: NR_104788.1
Ignicoccus hospitalis	NCBI Reference Sequence: NR_028955.1
Ignicoccus islandicus	NCBI Reference Sequence: NR_044910.1
Ignicoccus pacificus	GenBank: AJ271794.1
Impatiens walleriana	GenBank: L49285.1
Ipomoea batatas	GenBank: HM053485.1
Isochrysis galbana	GenBank: AJ246266.1
Isosphaera pallida	NCBI Reference Sequence: NR_028892.1
Klebsiella oxytoca	GenBank: AF390083.1
Klebsiella pneumoniae	GenBank: KC990817.1
Koliella antarctica	GenBank: AJ311569.1
Lactobacillus acidophilus	GenBank: KC150145.1
Lactobacillus delbrueckii	GenBank: KJ868760.1
Lactobacillus paracasei	NCBI Reference Sequence: NR_121787.1
Lactobacillus rhamnosus	NCBI Reference Sequence: NR_043408.1
Lactococcus lactis	GenBank: KR604712.1
Lactococcus piscium	GenBank: JN226414.1
Lactuca sativa	GenBank: KT225377.1
Lantana camara	GenBank: AJ236049.1
Larix decidua	GenBank: AB026938.1
Larrea tridentata	GenBank: AY929372.1
Lauderia annulata	GenBank: DQ514849.1
Lemna minor	GenBank: S67398.1
Lepidodinium chlorophorum	GenBank: AB686253.1
Leptocylindrus danicus	GenBank: AJ535175.1
Leptospirillum ferriphilum	GenBank: AF356830.1
Leptospirillum ferrooxidans	NCBI Reference Sequence: NR_074963.1
Limnothrix redekei	GenBank: FM177493.1
Lingulodinium polyedrum	GenBank: AB693195.1
Liriodendron tulipifera	GenBank: AF206954.1
Listeria monocytogenes	GenBank: M58822.1
Lithophyllum margaritae	GenBank: KP192392.1
Lolium multiflorum	GenBank: AY846367.1
Lolium perenne	GenBank: AY519271.1
Marinithermus hydrothermalis	NCBI Reference Sequence: NR_028639.1
Marinitoga piezophila	NCBI Reference Sequence: NR_074102.1
Marinobacter alkaliphilus	GenBank: EU440994.1
Mastigocladus laminosus	GenBank: DQ431003.1
Merismopedia tenuissima	GenBank: AJ639891.1
Mesotaenium kramstae	GenBank: AJ553922.1
Methanobacterium subterraneum	GenBank: JQ268007.1
Methanobacterium thermoaggregans	GenBank: AF095264.1
Methanobacterium thermoautotrophicum	GenBank: AF095262.1
Methanococcus jannaschii	GenBank: M59126.1
Methanococcus thermolithotrophicus	GenBank: M59128.1
Methanococcus voltae	NCBI Reference Sequence: NR_074184.1
Methanococcus vulcanius	NCBI Reference Sequence: NR_028701.1
Methanoculleus submarinus	NCBI Reference Sequence: NR_028856.1

Table D – *Continued from previous page*

Species	Accession ID
Methanogenium frigidum	NCBI Reference Sequence: NR_104790.1
Methanogenium frittonii	GenBank: AJ862839.1
Methanohalophilus portucalensis	GenBank: KT285318.1
Methanolobus psychrophilus	GenBank: EF202842.1
Methanopyrus kandleri	NCBI Reference Sequence: NR_074539.1
Methanosarcina barkeri	GenBank: M59144.1
Methanothermobacter thermautotrophicus	GenBank: DQ657903.1
Methanothermococcus okinawensis	NCBI Reference Sequence: NR_028155.1
Methanothrix soehngenii	NCBI Reference Sequence: NR_028242.1
Micrasterias americana	GenBank: FR852595.1
Microcystis aeruginosa	NCBI Reference Sequence: NR_074314.1
Microcystis wesenbergii	GenBank: U40334.1
Moritella abyssi	GenBank: AB554718.1
Moritella profunda	NCBI Reference Sequence: NR_025381.1
Mucuna pruriens	GenBank: AF525695.1
Mychonastes homosphaera	GenBank: X73996.1
Nannochloropsis oceanica	GenBank: FJ896231.1
Natrialba asiatica	NCBI Reference Sequence: NR_113519.1
Natrinema pellirubrum	NCBI Reference Sequence: NR_113528.1
Natronobacterium gregoryi	NCBI Reference Sequence: NR_113531.1
Natronococcus occultus	NCBI Reference Sequence: NR_113534.1
Natronomonas pharaonis	NCBI Reference Sequence: NR_113497.1
Natronorubrum bangense	NCBI Reference Sequence: NR_113538.1
Navicula arenaria	GenBank: KJ961668.1
Navicula pelliculosa	GenBank: AY485454.1
Nerium oleander	GenBank: AF107572.1
Nicotiana tabacum	GenBank: AJ236016.1
Nitrosotalea devanaterra	GenBank: JN227488.1
Nitzschia dissipata	GenBank: AJ867018.1
Nitzschia frigida	GenBank: JQ582669.1
Nitzschia paleacea	GenBank: AJ866996.1
Nitzschia sigma	GenBank: AJ867279.1
Odontella aurita	GenBank: HQ912687.1
Odontella mobiliensis	GenBank: KC309500.1
Odontella regia	GenBank: KC309502.1
Odontella sinensis	GenBank: HQ912564.1
Olea europaea	GenBank: L49289.1
Olisthodiscus luteus	GenBank: AY788937.1
Oryza sativa	GenBank: AF069218.1
Oscillatoria mougeotii	GenBank: FJ434250.1
Ostreopsis ovata	GenBank: AF244939.1
Palaeococcus helgesonii	NCBI Reference Sequence: NR_029059.1
Pandorina morum	GenBank: JQ315554.1
Papaver somniferum	GenBank: DQ912867.1
Paracoccus halodenitrificans	NCBI Reference Sequence: NR_025890.1
Paramecium tetraurelia	GenBank: EF502045.1
Paraphysomonas imperforata	GenBank: EF432519.1
Pavlova lutheri	GenBank: AF102369.1
Pediastrum duplex	GenBank: M62997.1
Pelagomonas calceolata	GenBank: EF455763.1
Pelotomaculum thermopropionicum	NCBI Reference Sequence: NR_040840.1
Peptostreptococcus productus	NCBI Reference Sequence: NR_113270.1

Table D – *Continued from previous page*

**Species**	**Accession ID**
Peridinium cinctum	GenBank: AB185114.1
Persephonella guaymasensis	NCBI Reference Sequence: NR_025166.1
Persephonella marina	NCBI Reference Sequence: NR_102828.1
Phaeocystis antarctica	GenBank: JN381495.1
Phaeocystis globosa	GenBank: AY851301.1
Phaeocystis pouchetii	GenBank: AJ278036.1
Phaeodactylum tricornutum	GenBank: GQ452861.1
Phyllogigas grandifolius	GenBank: HE866931.1
Picea mariana	GenBank: L01782.1
Picrophilus oshimae	NCBI Reference Sequence: NR_026246.1
Pinus elliottii	GenBank: AF051798.1
Pinus taeda	GenBank: AH001728.2
Pisum sativum	GenBank: U43011.1
Planktothrix agardhii	GenBank: FJ159128.1
Planococcus halocryophilus	GenBank: JF742665.1
Plantago lanceolata	GenBank: AJ236046.1
Populus tremuloides	GenBank: AF206999.1
Porphyra perforata	GenBank: GU319856.1
Porphyra umbilicalis	GenBank: AH010576.2
Porphyridium purpureum	GenBank: AB045584.1
Posidonia australis	GenBank: GQ497582.1
Posidonia oceanica	GenBank: AY491942.1
Potamogeton perfoliatus	GenBank: AY952389.1
Proboscia indica	GenBank: AY485470.1
Prochlorococcus marinus	NCBI Reference Sequence: NR_028762.1
Profundimonas piezophila	NCBI Reference Sequence: NR_117943.1
Prorocentrum concavum	GenBank: Y16237.1
Prorocentrum dentatum	GenBank: AY551273.1
Prorocentrum gracile	GenBank: AY443019.1
Prorocentrum lima	GenBank: Y16235.1
Prorocentrum mexicanum	GenBank: Y16232.1
Prorocentrum micans	GenBank: AJ415519.1
Prorocentrum minimum	GenBank: JF715165.1
Prunus persica	GenBank: L28749.1
Prymnesium polylepis	GenBank: AJ004866.1
Pseudoalteromonas antarctica	NCBI Reference Sequence: NR_029317.1
Pseudoalteromonas haloplanktis	GenBank: EU807989.1
Pseudochattonella verruculosa	GenBank: AM075625.1
Pseudomonas aeruginosa	GenBank: AM419153.2
Pseudomonas fluorescens	GenBank: AY538263.1
Pseudomonas putida	GenBank: KF278708.1
Pseudo-nitzschia fraudulenta	GenBank: JN091721.1
Pseudo-nitzschia granii	GenBank: GU373962.1
Pseudo-nitzschia multiseries	GenBank: AM235382.1
Pseudo-nitzschia pseudodelicatissima	GenBank: GU373965.1
Pseudo-nitzschia seriata	GenBank: GU373969.1
Pseudoxanthomonas broegbernensis	NCBI Reference Sequence: NR_025306.1
Pseudoxanthomonas taiwanensis	NCBI Reference Sequence: NR_025198.1
Psychrobacter glacincola	GenBank: AB334769.1
Psychrobacter muriicola	NCBI Reference Sequence: NR_114669.1
Psychroflexus torquis	GenBank: DQ007442.1
Psychromonas profunda	NCBI Reference Sequence: NR_025506.1

Table D – *Continued from previous page*

**Species**	**Accession ID**
Pyrobaculum aerophilum	GenBank: L07510.1
Pyrobaculum calidifontis	NCBI Reference Sequence: NR_040922.1
Pyrobaculum islandicum	NCBI Reference Sequence: NR_074372.1
Pyrobaculum oguniense	NCBI Reference Sequence: NR_112094.1
Pyrobaculum organotrophum	NCBI Reference Sequence: NR_112158.1
Pyrococcus abyssi	NCBI Reference Sequence: NR_115145.1
Pyrococcus furiosus	NCBI Reference Sequence: NR_074375.1
Pyrococcus glycovorans	NCBI Reference Sequence: NR_029053.1
Pyrococcus horikoshii	NCBI Reference Sequence: NR_115653.1
Pyrodinium bahamense	GenBank: DQ500120.1
Pyrolobus fumarii	NCBI Reference Sequence: NR_102985.1
Quercus rubra	GenBank: AF132892.1
Quercus suber	GenBank: GU476438.1
Ranunculus acris	GenBank: AH001745.2
Rhizosolenia robusta	GenBank: AY485481.1
Rhizosolenia setigera	GenBank: AY485461.1
Rhodomonas salina	GenBank: HM126532.1
Rosa hybrida	GenBank: X66773.1
Roya anglica	GenBank: AJ428081.1
Rubrobacter radiotolerans	GenBank: U65647.1
Rubrobacter xylanophilus	NCBI Reference Sequence: NR_074552.1
Ruppia maritima	GenBank: JN034103.1
Salmonella enterica	GenBank: KF535115.1
Scenedesmus acuminatus	GenBank: AB037088.1
Scenedesmus acutus	GenBank: AJ249512.1
Scenedesmus quadricauda	GenBank: KC790429.1
Scrippsiella trochoidea	GenBank: EF492513.1
Selenastrum minutum	GenBank: AY846380.1
Serratia marcescens	GenBank: GU991997.1
Setaria italica	GenBank: KC996746.1
Shewanella gelidimarina	GenBank: AY771753.1
Skeletonema ardens	GenBank: DQ396522.1
Skeletonema costatum	GenBank: JF489959.1
Skeletonema japonicum	GenBank: DQ011160.1
Skeletonema marinoi	GenBank: JF489953.1
Skeletonema menzelii	GenBank: AJ535168.1
Skeletonema pseudocostatum	GenBank: X85393.1
Skeletonema tropicum	GenBank: EF138941.1
Solanum lycopersicum	GenBank: KJ813729.1
Solanum tuberosum	GenBank: FJ710157.1
Solenostemon scutellarioides	GenBank: EU019244.1
Sorghum bicolor	GenBank: AH001770.2
Sphaerospermopsis aphanizomenoides	GenBank: GU197654.1
Sphagnum angustifolium	GenBank: GQ375058.1
Sphagnum squarrosum	GenBank: GQ375075.1
Spinacia oleracea	GenBank: L24420.1
Spiroplasma apis	NCBI Reference Sequence: NR_104858.1
Spiroplasma cantharicola	NCBI Reference Sequence: NR_125516.1
Spiroplasma chinense	NCBI Reference Sequence: NR_025698.1
Spiroplasma citri	NCBI Reference Sequence: NR_036849.1
Spiroplasma clarkii	NCBI Reference Sequence: NR_104750.1
Spiroplasma culicicola	NCBI Reference Sequence: NR_025701.1

Table D – *Continued from previous page*

**Species**	**Accession ID**
Spiroplasma diminutum	NCBI Reference Sequence: NR_025702.1
Spiroplasma floricola	NCBI Reference Sequence: NR_025703.1
Spiroplasma insolitum	NCBI Reference Sequence: NR_025705.1
Spiroplasma ixodetis	NCBI Reference Sequence: NR_104852.1
Spiroplasma kunkelii	NCBI Reference Sequence: NR_104847.1
Spiroplasma mirum	NCBI Reference Sequence: NR_118707.1
Spiroplasma monobiae	NCBI Reference Sequence: NR_104854.1
Spiroplasma sabaudiense	NCBI Reference Sequence: NR_025710.1
Spiroplasma taiwanense	NCBI Reference Sequence: NR_121701.1
Spiroplasma velocicrescens	NCBI Reference Sequence: NR_025713.1
Spirulina platensis	GenBank: AB074508.1
Staphylococcus aureus	GenBank: DQ630753.1
Staphylococcus xylosus	NCBI Reference Sequence: NR_036907.1
Staurastrum avicula	GenBank: EF507555.1
Staurastrum pingue	GenBank: AJ428109.1
Staurodesmus cuspidatus	GenBank: EF507538.1
Stellarima microtrias	GenBank: EU090011.1
Stephanodiscus hantzschii	GenBank: DQ093370.1
Stephanopyxis palmeriana	GenBank: AY485527.1
Streptococcus salivarius	GenBank: M58839.1
Streptococcus thermophilus	GenBank: AB812892.1
Stygiolobus azoricus	NCBI Reference Sequence: NR_043434.1
Sulfobacillus benefaciens	GenBank: EF679212.1
Sulfobacillus sibiricus	NCBI Reference Sequence: NR_042730.1
Sulfobacillus thermosulfidooxidans	GenBank: EU499919.1
Sulfobacillus thermotolerans	GenBank: JX966410.1
Sulfolobus acidocaldarius	NCBI Reference Sequence: NR_043400.1
Sulfolobus hakonensis	NCBI Reference Sequence: NR_028222.1
Sulfolobus metallicus	NCBI Reference Sequence: NR_043433.1
Sulfolobus tengchongensis	NCBI Reference Sequence: NR_115150.1
Sulfolobus yangmingensis	NCBI Reference Sequence: NR_028603.1
Sulfophobococcus zilligii	NCBI Reference Sequence: NR_029316.1
Sulfurihydrogenibium kristjanssoni	GenBank: AM778960.1
Sulfurisphaera ohwakuensis	NCBI Reference Sequence: NR_043432.1
Symbiobacterium toebii	GenBank: AF190460.1
Symbiodinium microadriaticum	GenBank: KU900226.1
Synechococcus elongatus	GenBank: HF678511.1
Synechococcus lividus	GenBank: AF132772.1
Syntrophothermus lipocalidus	NCBI Reference Sequence: NR_040796.1
Thalassionema nitzschioides	GenBank: X77702.2
Thalassiosira allenii	GenBank: HM991688.1
Thalassiosira constricta	GenBank: KT692951.1
Thalassiosira curviseriata	GenBank: AJ810859.1
Thalassiosira eccentrica	GenBank: X85396.1
Thalassiosira guillardii	GenBank: AF374478.2
Thalassiosira hendeyi	GenBank: AM050629.1
Thalassiosira nordenskioeldii	GenBank: DQ093365.1
Thalassiosira pseudonana	GenBank: AY485452.1
Thalassiosira rotula	GenBank: AF374480.2
Thalassiosira weissflogii	GenBank: AY485445.1
Thermacetogenium phaeum	NCBI Reference Sequence: NR_074723.1
Thermaerobacter nagasakiensis	NCBI Reference Sequence: NR_024776.1

Table D – *Continued from previous page*

Species	Accession ID
Thermoanaerobacter ethanolicus	NCBI Reference Sequence: NR_044619.1
Thermoanaerobacter keratinophilus	NCBI Reference Sequence: NR_115188.1
Thermoanaerobacter mathranii	GenBank: LC127101.1
Thermoanaerobacter siderophilus	GenBank: KR736354.1
Thermoanaerobacter subterraneus	GenBank: EU109461.1
Thermoanaerobacter tengcongensis	GenBank: AF209708.1
Thermoanaerobacter yonseiensis	GenBank: HM228410.1
Thermobrachium celere	GenBank: DQ207958.2
Thermococcus alcaliphilus	NCBI Reference Sequence: NR_040870.1
Thermococcus barophilus	NCBI Reference Sequence: NR_042734.1
Thermococcus barossii	NCBI Reference Sequence: NR_042735.1
Thermococcus celer	NCBI Reference Sequence: NR_042736.1
Thermococcus chitonophagus	NCBI Reference Sequence: NR_119236.1
Thermococcus fumicolans	NCBI Reference Sequence: NR_042738.1
Thermococcus hydrothermalis	NCBI Reference Sequence: NR_042740.1
Thermococcus peptonophilus	NCBI Reference Sequence: NR_028193.1
Thermococcus siculi	NCBI Reference Sequence: NR_028195.1
Thermocrinis ruber	NCBI Reference Sequence: NR_121741.1
Thermodesulfovibrio yellowstonii	NCBI Reference Sequence: NR_074345.1
Thermoplasma acidophila	NCBI Reference Sequence: NR_028235.1
Thermoproteus uzoniensis	NCBI Reference Sequence: NR_102955.1
Thermosipho japonicus	NCBI Reference Sequence: NR_024726.1
Thermosphaera aggregans	NCBI Reference Sequence: NR_074380.1
Thermosyntropha lipolytica	NCBI Reference Sequence: NR_026356.1
Thermoterrabacterium ferrireducens	GenBank: U76364.1
Thermotoga lettingae	NCBI Reference Sequence: NR_074951.1
Thermotoga maritima	NCBI Reference Sequence: NR_029163.1
Thermus aquaticus	NCBI Reference Sequence: NR_025900.1
Thermus chliarophilus	NCBI Reference Sequence: NR_026244.1
Thermus thermophilus	NCBI Reference Sequence: NR_037066.1
Trichococcus patagoniensis	GenBank: AF394926.1
Trichodesmium erythraeum	NCBI Reference Sequence: NR_074275.1
Trifolium repens	GenBank: AF071069.1
Triticum aestivum	GenBank: AY049040.1
Tychonema bourrellyi	GenBank: FJ184385.1
Ulva lactuca	GenBank: KF419328.1
Vallisneria americana	GenBank: AF069201.1
Veratrum californicum	GenBank: AH003503.2
Vibrio marinus	GenBank: AJ297540.1
Vitis vinifera	GenBank: GQ849399.1
Volvox aureus	GenBank: LC086362.1
Xanthomonas campestris	GenBank: AF290420.1
Xylella fastidiosa	NCBI Reference Sequence: NR_115924.1
Yersinia enterocolitica	GenBank: M59292.1
Zea mays	GenBank: AF168884.1
Zostera marina	GenBank: HQ445940.1
Zostera noltii	GenBank: AF207058.1

Table E. Species names and Accession IDs of cbbL/rbcL gene sequences that were used in this study.

Species	Accession ID
Abies alba	GenBank: AB029652.1

Table E – *Continued from previous page*

Species	Accession ID
Abutilon theophrasti	GenBank: HM849734.1
Acer rubrum	GenBank: DQ978428.1
Acidimicrobium ferrooxidans	GenBank: GQ409765.1
Acidithiobacillus caldus	GenBank: GQ409763.1
Acidithiobacillus ferrivorans	GenBank: FJ467341.1
Acidithiobacillus ferrooxidans	GenBank: GQ409767.1
Acidithiobacillus thiooxidans	GenBank: GQ225727.1
Aldrovanda vesiculosa	GenBank: AY096106.1
Amphiprora paludosa	GenBank: FJ002140.1
Anabaena ucrainica	GenBank: GU197741.1
Antirrhinum majus	GenBank: GQ997015.1
Aphanizomenon ovalisporum	GenBank: KP698018.1
Aplectrum hyemale	GenBank: AF074108.1
Arabidopsis thaliana	GenBank: KU739560.1
Arbutus unedo	GenBank: KF997388.1
Aristotelia serrata	GenBank: AF307904.1
Asterionella formosa	GenBank: HQ912497.1
Asterionellopsis glacialis	GenBank: HQ912510.1
Aulacoseira granulata	GenBank: AB430659.1
Beta vulgaris	GenBank: KM360669.1
Betula papyrifera	GenBank: X56617.1
Betula pendula	GenBank: KM360670.1
Brassica oleracea	GenBank: GQ184376.1
Brassica rapa	GenBank: KJ473492.1
Bryum argenteum	GenBank: AY163024.1
Capsella bursa-pastoris	GenBank: KT458036.1
Capsicum annuum	GenBank: KJ773334.1
Carya glabra	GenBank: L12637.2
Caulerpa serrulata	GenBank: JQ745697.1
Ceratophyllum demersum	GenBank: AB917052.1
Chamaebatiaria millefolium	GenBank: U06797.1
Chamerion angustifolium	GenBank: L10217.1
Chenopodium album	GenBank: JX848451.1
Chlamydomonas reinhardtii	GenBank: AB511846.1
Chlamydomonas subcaudata	GenBank: GQ871929.1
Chlorella ellipsoidea	GenBank: EU038287.1
Chlorella pyrenoidosa	GenBank: EU038283.1
Chlorella saccharophila	GenBank: AM260446.1
Chlorella sorokiniana	GenBank: HM101339.1
Chlorella vulgaris	GenBank: EU038286.1
Chondrus crispus	GenBank: U02984.1
Chrysanthemum morifolium	GenBank: KM218356.1
Cicer arietinum	GenBank: AF308707.1
Citrus aurantium	GenBank: AB505953.1
Citrus limon	GenBank: AB505956.1
Closterium acerosum	GenBank: AF203492.1
Coccolithus pelagicus	GenBank: HQ656833.1
Coelastrum microporum	GenBank: KP698027.1
Coscinodiscus concinnus	GenBank: HQ912545.1
Coscinodiscus granii	GenBank: HQ656838.1
Coscinodiscus jonesianus	GenBank: KJ577887.1
Cosmarium biretum	GenBank: AM911267.1

Table E – *Continued from previous page*

Species	Accession ID
Cosmarium botrytis	GenBank: AM911295.1
Cosmarium crenatum	GenBank: AM911268.1
Cosmarium meneghinii	GenBank: AM911284.1
Cosmarium punctulatum	GenBank: AM911289.1
Cosmarium subprotumidum	GenBank: AM911292.1
Cryptomonas marssonii	GenBank: AM051209.1
Cryptomonas ovata	GenBank: AM051211.1
Cucumis sativus	GenBank: L21937.1
Cyclotella cryptica	GenBank: KM816805.1
Cyclotella meneghiniana	GenBank: KM816803.1
Cylindrospermopsis raciborskii	GenBank: JF895153.1
Cylindrotheca closterium	GenBank: JX971010.1
Cymodocea nodosa	GenBank: U80688.1
Daucus carota	GenBank: KM360751.1
Desmarestia anceps	GenBank: HE866816.1
Desmidium swartzii	GenBank: HQ380525.1
Detonula confervacea	GenBank: HQ912481.1
Diapensia lapponica	GenBank: L12612.2
Ditylum brightwellii	GenBank: DQ514766.1
Dunaliella tertiolecta	GenBank: JQ039069.1
Egeria densa	GenBank: AB004887.1
Elodea canadensis	GenBank: DQ859167.1
Emiliania huxleyi	GenBank: JX292160.1
Enteromorpha intestinalis	GenBank: AF499671.1
Eucalyptus globulus	GenBank: HM849985.1
Eucampia zodiacus	GenBank: KC309568.1
Eucheuma isiforme	GenBank: AF099691.1
Fibrocapsa japonica	GenBank: AB280606.1
Fontinalis antipyretica	GenBank: AB050949.1
Fragilaria crotonensis	GenBank: HQ828187.2
Fragilariopsis cylindrus	GenBank: EF423499.1
Fragilariopsis kerguelensis	GenBank: KC920826.1
Gephyrocapsa oceanica	GenBank: D45844.1
Gerbera jamesonii	GenBank: L13643.1
Glycine max	GenBank: Z95552.1
Gonatozygon monotaenium	GenBank: FM992338.1
Gossypium hirsutum	GenBank: JQ034248.1
Gracilaria verrucosa	GenBank: JQ843364.1
Grammonema striatula	GenBank: KF701600.1
Guinardia flaccida	GenBank: KC309609.1
Gymnodinium breve	GenBank: AY119786.1
Gymnodinium mikimotoi	GenBank: JX899690.2
Haematococcus pluvialis	GenBank: FJ438476.1
Helianthus annuus	GenBank: L13929.1
Heterosigma akashiwo	GenBank: HQ710629.1
Hordeum vulgare	GenBank: LN626641.1
Hormidium flaccidum	GenBank: EU477433.1
Hydrilla verticillata	GenBank: KM982379.1
Impatiens walleriana	GenBank: AB043508.1
Ipomoea batatas	GenBank: JQ923431.1
Isochrysis galbana	GenBank: HQ656829.1
Lactuca sativa	GenBank: AY874437.1

Table E – *Continued from previous page*

**Species**	**Accession ID**
Lantana camara	GenBank: HM850104.1
Larix decidua	GenBank: FN689379.1
Larrea tridentata	GenBank: Y15022.1
Lauderia annulata	GenBank: DQ514769.1
Lemna minor	GenBank: AM905730.1
Lepidodinium chlorophorum	GenBank: AY331683.1
Leptocylindrus danicus	GenBank: JX413575.1
Liriodendron tulipifera	GenBank: AF190430.1
Lolium multiflorum	GenBank: LT576830.1
Lolium perenne	GenBank: AY395547.1
Mastigocladus laminosus	GenBank: JQ918781.1
Mesotaenium kramstae	GenBank: AJ553952.1
Microcystis aeruginosa	GenBank: KP698052.1
Mucuna pruriens	GenBank: EU128733.1
Mychonastes homosphaera	GenBank: KC145515.1
Nannochloropsis oceanica	GenBank: KT149178.1
Navicula pelliculosa	GenBank: HQ337547.1
Nerium oleander	GenBank: HM850198.1
Nicotiana tabacum	GenBank: KC825342.1
Odontella aurita	GenBank: HQ912551.1
Odontella mobiliensis	GenBank: KC309574.1
Odontella regia	GenBank: KC309576.1
Odontella sinensis	GenBank: HQ912428.1
Olea europaea	GenBank: DQ673304.1
Olisthodiscus luteus	GenBank: AB280605.1
Oryza sativa	GenBank: AJ746297.1
Pandorina morum	GenBank: AB044165.1
Papaver somniferum	GenBank: HM850232.1
Pavlova lutheri	GenBank: HQ656830.1
Pediastrum duplex	GenBank: EF078364.1
Pelagomonas calceolata	GenBank: U89898.1
Phaeocystis antarctica	GenBank: KP144261.1
Phaeocystis globosa	GenBank: HQ656835.1
Phaeodactylum tricornutum	GenBank: HQ912420.1
Phyllogigas grandifolius	GenBank: HE866853.1
Picea mariana	GenBank: EU364784.1
Pinus elliottii	GenBank: AB081075.1
Pinus taeda	GenBank: AF119177.1
Pisum sativum	GenBank: JN661190.1
Planktothrix agardhii	GenBank: EU151930.1
Plantago lanceolata	GenBank: L36454.1
Populus tremuloides	GenBank: AF206812.1
Porphyra perforata	GenBank: AF452438.1
Porphyra umbilicalis	GenBank: AF452446.1
Porphyridium purpureum	GenBank: DQ308439.1
Posidonia australis	GenBank: U80718.1
Posidonia oceanica	GenBank: U80719.1
Potamogeton perfoliatus	GenBank: AY952437.1
Proboscia indica	GenBank: JQ315456.1
Prochlorococcus marinus	GenBank: AY042090.1
Prunus persica	GenBank: AF411493.1
Pseudochattonella verruculosa	GenBank: AB280607.1

Table E – *Continued from previous page*

Species	Accession ID
Pseudo-nitzschia fraudulenta	GenBank: EF423503.1
Pseudo-nitzschia granii	GenBank: KU183494.1
Pseudo-nitzschia multiseries	GenBank: KC801040.1
Pseudo-nitzschia pseudodelicatissima	GenBank: KC801039.1
Quercus rubra	GenBank: LM653089.1
Quercus suber	GenBank: LM653092.1
Ranunculus acris	GenBank: KF602170.1
Rhizosolenia robusta	GenBank: JQ315467.1
Rhizosolenia setigera	GenBank: HQ912425.1
Roya anglica	GenBank: AJ553963.1
Ruppia maritima	GenBank: HQ901576.1
Scenedesmus quadricauda	GenBank: AB084332.1
Setaria italica	GenBank: EF125138.1
Skeletonema ardens	GenBank: JN162820.1
Skeletonema costatum	GenBank: AF015569.1
Skeletonema japonicum	GenBank: DQ514822.1
Skeletonema marinoi	GenBank: KJ671816.1
Skeletonema menzellii	GenBank: DQ514821.1
Skeletonema pseudocostatum	GenBank: DQ514819.1
Skeletonema tropicum	GenBank: KJ671817.1
Solanum lycopersicum	GenBank: HF572813.1
Solanum tuberosum	GenBank: KJ652187.1
Sorghum bicolor	GenBank: AM849341.1
Sphaerospermopsis aphanizomenoides	GenBank: FJ830541.1
Sphagnum angustifolium	GenBank: AY309690.1
Sphagnum squarrosum	GenBank: AY309706.1
Spirulina platensis	GenBank: AY147205.1
Staurastrum pingue	GenBank: AF203506.1
Stellarima microtrias	GenBank: EU090032.1
Stephanodiscus hantzschii	GenBank: AB831882.1
Stephanopyxis palmeriana	GenBank: KP253080.1
Sulfobacillus thermosulfidooxidans	GenBank: GQ409769.1
Thalassionema nitzschioides	GenBank: KJ671820.1
Thalassiosira constricta	GenBank: KT692950.1
Thalassiosira curviseriata	GenBank: KJ671821.1
Thalassiosira eccentrica	GenBank: DQ514789.1
Thalassiosira guillardii	GenBank: DQ514796.1
Thalassiosira nordenskioeldii	GenBank: KC985865.1
Thalassiosira pseudonana	GenBank: HQ912419.1
Thalassiosira rotula	GenBank: DQ514805.1
Thalassiosira weissflogii	GenBank: DQ514811.1
Trichodesmium erythraeum	GenBank: AB075924.1
Trifolium repens	GenBank: KF602192.1
Triticum aestivum	GenBank: LT576864.1
Ulva lactuca	GenBank: EU484409.1
Vallisneria americana	GenBank: U03726.1
Vitis vinifera	GenBank: AJ635355.1
Volvox aureus	GenBank: D63445.1
Zea mays	GenBank: Z11973.1
Zostera marina	GenBank: AB125348.1
Zostera noltii	GenBank: U80733.1

References

[1] Stamatakis A. RAxML version 8: a tool for phylogenetic analysis and post-analysis of large phylogenies. Bioinformatics. 2014;30(9):1312–1313.

[2] Heath TA, Holder MT, Huelsenbeck JP. A Dirichlet process prior for estimating lineage-specific substitution rates. Mol Biol Evol. 2012;29(3):939–955.

[3] Hinchliff CE, Smith SA, Allman JF, Burleigh JG, Chaudhary R, Coghill LM, et al. Synthesis of phylogeny and taxonomy into a comprehensive tree of life. P Natl Acad Sci USA. 2015;112(41):12764–12769.

[4] Elliot MG, Mooers AØ. Inferring ancestral states without assuming neutrality or gradualism using a stable model of continuous character evolution. BMC Evol Biol. 2014;14(1):226.

[5] Mooers AØ, Vamosi SM, Schluter D. Using phylogenies to test macroevolutionary hypotheses of trait evolution in cranes (Gruinae). Am Nat. 1999;154(2):249–259.

[6] Duchen P, Leuenberger C, Szilágyi SM, Harmon L, Eastman J, Schweizer M, et al. Inference of evolutionary jumps in large phylogenies using Lévy processes. Syst Biol. 2017;66(6):950–963.

[7] Jenks GF. The data model concept in statistical mapping. International yearbook of cartography. 1967;7:186–190.

[8] Rabosky DL, Grundler M, Anderson C, Title P, Shi JJ, Brown JW, et al. BAMMtools: an R package for the analysis of evolutionary dynamics on phylogenetic trees. Methods Ecol Evol. 2014;5:701–707.

[9] Pagel M. Detecting correlated evolution on phylogenies: a general method for the comparative analysis of discrete characters. Proc Biol Sci. 1994;255(1342):37–45.

[10] Revell LJ. phytools: an R package for phylogenetic comparative biology (and other things). Methods Ecol Evol. 2012;3(2):217–223.
