# Supplementary material for: Adaptive evolution shapes the present-day distribution of the thermal sensitivity of population growth rate
Source: PLoS Biol. 2020 Oct 16;18(10):e3000894. doi: 10.1371/journal.pbio.3000894 (PMC7592915; doi:10.1371/journal.pbio.3000894)
Supplement: Attachment — Submitted filename: Kontopoulos_etal_Rev_Responses.pdf [file pbio.3000894.s002.pdf]

Responses to the Reviewers

Comments made by Reviewer #1, #2, and #3 are in **green**, **dark orange**, and **purple** respectively whereas our responses are in **black**.

Comments by Reviewer #1

1. The research presented here aims to answer two questions: How variation in thermal sensitive accumulates across multiple autotroph and heterotroph groups (meaning, over time?), and then, whether variation in thermal sensitivity is shaped by environmental selection. This is an important question and of great interest to scientists across disciplines of biology, from physiology to ecology, and the results here are certainly novel and exciting. Variation among thermal sensitivities among taxa has certainly been demonstrated before, and so has variation in temperature dependence of metabolic rates among prokaryote and eukaryote taxonomic groups (e.g., White et al Proc Roy Soc 2012 279: 1742, Galmes et al, Photosynthesis research 2015: 123(2) 183-201.) But, a robust analysis explicitly testing hypotheses from MTE about the universality of activation energy parameters that quantitatively considers phylogenies lacking, to my knowledge. Therefore, I believe this study constitutes a substantial and important contribution to the literature. My belief would be confirmed if the authors could explicitly relate their findings to the two studies noted above (and others if I've missed them) that reach similar findings by different methods (White et al) or different findings (Galmes et al) than reported in the present study.

   Thank you for your kind words. These two studies indeed present evidence for variation in thermal sensitivity across taxonomic groups, which suggests that thermal sensitivity may be a phylogenetically heritable trait as we show in the present study. We now cite the study by White et al. in the Introduction (line 48) and in the Discussion (line 455), when we refer to studies that identified taxonomically-structured variation in thermal sensitivity. We also now compare our findings with those of Galmés et al., about adaptive variation in the TPC of Rubisco carboxylase specific activity (lines 427–429).

2. **All of the following comments are grouped together because they pertain to the same topic: hypotheses tested in this study.**

   As I mentioned already, I think the findings of this paper are of general interest, but the potential limitations or assumptions in the work are not clear to a non-specialist reader as the manuscript is currently written. I recommend making much more clear and explicit the set of hypotheses tested, and the assumptions inherent in the hypothesis as well as in the methods used to test them. I recommend revising the introduction to explicitly state a set of testable (rejectable) hypotheses. I find myself trying to figure out what the hypotheses are, but really it is the job of the authors to tell me. My reading of the introduction leads me to think that the hypotheses being tested here are:

   H0: there is no phylogenetic signal to thermal sensitivity traits (as defined by the empirically fit sharpe-schoolfield equation). This would be consistent with MTE's UTD in its simplest form. (The authors refer to this as an MTE assumption - line 89.)

   One prediction associated with this hypothesis that the authors have given is that measurement error could explain any patterns in the data, rather than phylogenetic signal. The introduction nicely explains what patterns would support or refute this prediction. I would like to see the alternative predictions introduced as clearly.

Another hypothesis I draw out of the introduction is:

H1: there is phylogenetic signal to thermal sensitivity traits, reflecting environmental conditions experienced by species or groups during their evolution. The authors could be more specific about what these patterns might be expected to be. Lines 184-186: "This high convergence in thermal sensitivity space by diverse lineages suggests that variation in the two TPC parameters is mainly driven by adaptation to local environmental conditions, irrespective of the species evolutionary history". Here is a place where the strength of inference and findings would be more clear if a (full?) set of alternate hypotheses were laid out clearly. From what is written, I infer that the possible interpretations for patterns of variation in TPC parameters across species are:

- Evolutionary history (presumably independent of the environmental influence?)
- Environmental conditions (independent of evolutionary history, as noted by the authors)
- Measurement error (reject above)
- What are other possibilities - constraints on possible traits associated with physical processes (rather than genetic constraints associated with evolution)? Can alternatives be rejected? Is an interaction between evolutionary history and environment possible, and if so, how would that be diagnosed?

The first paragraph of the discussion references two hypotheses, and it would be stronger to see these set up more obviously in the introduction. It is there, but the writing in that first paragraph of the discussion is much more clear in terms of presenting the hypotheses tested.

We agree. The reviewer has correctly reconstructed most of our (previously implicit) hypotheses. We now explicitly state and describe our hypotheses in lines 94–127 of the Introduction.

3. On the geographic variation hypotheses (H2, perhaps), there are alternate explanations that should be considered (especially because thermal variation (annual?) as a predictor was not explicitly tested or even shown. Furthermore, rationale should be given for how (annual?) thermal variation is expected to matter for organisms with such high rates of population growth and presumably evolution. Additionally, many plankton have resting stages in which their populations do not grow during harsh conditions. So the link between thermal variation and diversity of thermal traits in unicellular algae needs to be explained a bit more, and alternative need to be explored. Many ecological and evolutionary patterns and processes vary with latitude, other than thermal variation. Most notably, biodiversity and evolutionary history (tropical groups are generally older). These should be considered or at least mentioned. When this comes up in the discussion (line 279), one alternative explanation is considered (MCA). The finding that E varies with latitude but not W may (or may not) be consistent with explanations other than simply different sample sizes.

   The reviewer raises a very important point. We were not sufficiently explicit about the issue of time scales of environmental fluctuations, even though such fluctuations lie at the heart of our explanation for why E varies. Evolutionary theory suggests that variation within and across generations should select for different thermal sensitivity strategies [1]. The lower bound for generation time in microbes can be obtained by calculating the inverse of B_{pk} of r_{max}. Based on this, the minimum generation time across our microbial datasets ranges from 17.5 minutes to 3.5 months (see also Fig. S14). Thus, environmental

variability at intra-annual timescales should have significant impacts on species' E's across our dataset. Indeed, there is recent evidence that such effects can be seen relatively rapidly in the lab [2].

Nevertheless, a thorough examination of the issue of timescales of fluctuations is outside the scope of our study, because practically all our data come from populations that have been grown in the lab in non-fluctuating environments for at least a few generations after isolation from their (naturally fluctuating) environment. Therefore, microevolutionary (but *not* macro-evolutionary) changes in E driven by local environments may have possibly been reversed. At the same time, rarely did the original studies from where these data were extracted report the date of isolation of the population from its environment. Furthermore, there is no single reliable temperature dataset that covers the diversity of marine, freshwater, and terrestrial environments that our species/isolates represent. We have in fact explored fine-scale effects of thermal fluctuations in marine phytoplankton in a separate study, which illustrates the data limitations that make detection of effects of fine-scale fluctuations difficult [3].

In any case, at the macroevolutionary scale, we expect that our argument and hypothesis that it is fluctuations along latitudes that underlie the patterns of E variation is robust. This is because at that coarse scale, annual as well as intra-annual fluctuations in temperature at different timescales are strongly correlated [4,5]. We now explain how fluctuating environments impose different selection pressures for thermal sensitivity in lines 400-415.

Lastly, the fact that evolutionary history itself varies with latitude is an interesting one that we had not considered. We have now added an extra analysis that shows that phylogenetic distance does not correlate strongly (or at all) with latitudinal distance in phytoplankton and prokaryotes, the two main groups of our study (lines 316–324). This indicates that the association between E and latitude is not mainly due to a clade-age bias.

4. Line 204 - 206: This seems like an interesting result but needs more explanation and setup. Again, a set of hypotheses or predictions might help here. As a reader, I was unprepared for the possible outcomes of this test, and therefore it was unclear what were the implications of their actual finding. It would also help if, when introducing possible outcomes, the authors could highlight key assumptions of their methods and whether those assumptions might lend uncertainty to the conclusions. I think this would help a general biologist reader of PLoS biology who does not have expertise in phylogenetics (like myself).

   We now explicitly link this analysis to the hypotheses tested in this study (lines 118–123 and 243–244). Specifically, it could be possible that certain clades have higher evolutionary rates in thermal sensitivity, allowing them to better adapt to different environments (part of hypothesis 3). In contrast to this, we found that no systematic variation in evolutionary rate exists across clades.

5. The results and analysis in Figure 6 are hard to understand. It is not clear why variation in thermal sensitivity among taxa increases with time, nor how one can confidently reconstruct historical thermal sensitivities having previously concluded that they are not explained by evolutionary history but rather by environmental conditions. Does the reconstruction in figure 6 not assume that these traits are affected by evolution, and therefore contradict the previous result?

   To infer the ancestral states, we used the stable model of trait evolution. This model does not assume a constant evolutionary rate but allows for small or large jumps in the parameter space. Thus, this model can accommodate rapid shifts in trait values that

could be the outcome of environmental selection. The uncertainty for each ancestral state is shown in red and can, indeed, be substantial for many tree nodes (e.g., the root in panels A and C).

Finally, if thermal sensitivity evolved according to the MTE expectation, all lineages would be evolving very closely to the trait value at the root of the tree. Instead, Fig. 6 shows that lineages increasingly explore the parameter space of thermal sensitivity, without being strongly attracted close to the value at the root node. We now clarify this in lines 104–117.

6. Methods: Though I can't evaluate the phylogenetic methods, I do think the authors' general statistical approach of ensuring that their datasets do not include overlapping species, and the consideration of possible covariation among TPC parameters in their analysis are appropriate.

   How much confidence do the authors have in the phylogenetic estimates of time (their relative time axis)? These uncertainties should be explained. Not being an expert, I assume it is likely that evolutionary relationships are better resolved for more recent divisions compared to those in deeper time; could the uncertainty in phylogenies associated with time explain part of the results in figure 3?

   Indeed, accurate phylogenetic resolution is often much harder for nodes closer to the root of the tree, especially if the very first branching event occurred billions of years ago. It is for this reason that we used the Open Tree of Life (OTL) as a topology constraint, as it is based on many published phylogenies and represents our current best estimate of the tree of life. After applying the OTL constraints, the majority of nodes in the phylogeny had strong statistical support (i.e., a bootstrap value $\geq 75\%$; see Fig. S1) and thus the phylogeny is robust.

   Furthermore, Fig. 3 indicates that there is greater variation in thermal sensitivity within subclades than among them, especially near the present. In other words, thermal sensitivity tends to differ more between closely related species than between different clades (see also Fig. 4). Because phylogenetic resolution generally tends to be higher at nodes near the present (i.e., nodes that connect closely related species), we have no reason to believe that the result in Fig. 3 is a phylogenetic artefact. We now clarify this issue in lines 238–241.

7. Minor comments: There seems to be increasing evidence that the E value of photosynthesis may not universally be 0.32, as was suggested by Allen et al 2005. There has been support for this value, mostly in algae, but less in terrestrial plants. You might consider removing this sentence (line 29-31). However, this is very much unresolved. The work I've seen that convinced me that 0.32 is not well-supported is not yet published! So I simply suggest this edit; it's up to you to leave it or keep it.

   Two of the hypotheses that we test in this study are based on the Universal Temperature Dependence (UTD) concept of the MTE, according to which the E values for photosynthetic organisms should be close to 0.32 eV. This assumption serves as a "null expectation" in our study and, for this reason, we believe that it is important to mention and test it. This is now made more explicit in lines 94–111. Nevertheless, we do agree that the substantial variation in E (as shown by multiple studies including ours [6–8]) is in direct contrast to what would be expected under the UTD concept.

8. Another point that would increase clarity for a general reader would be to use the terms rather than the parameter names (or, in addition to). For example, on line 210, the sentence could refer to temperature dependence and thermal niche breadth rather than just E and W0.

We feel we should stick with parameter symbols throughout the text for brevity. However, we now state what E and W_{op} are in words in parentheses when these are first used in the Results and the Discussion sections.

Comments by Reviewer #2

9. Review of "Adaptive evolution shapes the present-day distribution of the thermal sensitivity of population growth rate"

   In this article, the authors undertake several analyses to determine whether features of thermal performance curves (TPCs) in phytoplankton and prokaryotes have/can evolve or are constrained by underlying thermodynamic constraints of biochemistry. Altogether, the authors conclude that, contrary to the hypothesis that there is universal temperature dependence (UTD), that activation energy (E, describing the increase in the left side of the TPC) has diverged through time and that different clades explore considerable variation in E within clades. They also show that much of this variation has occurred in more recent parts of their timelines. They make similar conclusions that TPC breadth (Wop) displays these same patterns, which are also consistent with the evolution of respiration and photosynthesis in plants, which can be considered underlying drivers of growth. Finally, they find that E declines with latitude for all types of TPCs in their analysis, but not so for Wop.

   OK, there is a lot going on in this article, so sorry it took a while. Overall, I think it's a great contribution. It provides fundamental insights into the evolution of thermal tolerance, not just retrospectively through the lens of phylogeny but also that further evolution of thermal sensitivity is possible. It's a novel take on a core problem. This paper should be the final death knell of the UTD as we currently think of it, not that there couldn't be other ways that thermodynamics constrain physiology. The paper is thorough, well-written, and the figures are engaging and informative.

   I do have some questions and comments, a few little things to annoy the authors with, but hopefully they will be useful.

   First, since these are most if not all (presumably) lab cultures, to what extent is adaptation/acclimation to a typical lab temperature influencing the perceived pattern of evolution? If over many generations the cultures have shifted their TPCs to adapt to ∼22C, as is clearly possible given (Padfield et al. 2016), then would the results reflect some convergence around the lab environment rather than the phylogenetic pathways that these organisms travel in the wild? I don't believe there is anything to be done about this possibility in the analysis, but it could be influencing the results.

   Thank you for your kind words. Our data typically come from species or strains which were isolated from the environment and were grown at a range of fixed temperatures after brief (but sufficient) periods of acclimation or even evolution over a few generations at least (also see our response to comment #3 above). We do not expect mutation-limited evolutionary changes in TPC shape over such short timescales. Furthermore, even when experimentalists maintain strains in the lab for prolonged periods, they do not incubate them at a common lab temperature, but set the temperature according to the preferences of each given strain (e.g., see Fig. 2 of Smith et al. 2019 [8]).

   However, culturing strains for hundreds of generations could indeed result in adaptation of TPCs to the laboratory conditions, as shown by e.g., Padfield et al. [9]. We now clarify

this in the Discussion (lines 446–451).

10. Abstract. Where it says (there are no line numbers here) 'typically-experienced thermal range', I would change this, because there is no clear reason why the rising portion of a curve is where most of the experienced temperatures are. Certainly many species can experience typical temperatures around the peak. Also see line 22 in the introduction. I would also say your results are 'consistent with' rather than 'driven by two opposing...' factors. Only if you could isolate the effect of temperature fluctuations explicitly (e.g., with experiments) could you go as far as 'driven'. Also here, evolution can occur at ecological time scales for anything; this is not a unique feature of microbes.

    Previous studies suggest that species tend to exhibit T_{pk} values that are greater than the mean environmental temperature [10–12]. We now make this clearer in lines 23–25 of the Introduction and in the caption of Fig. 1.

    Regarding the use of "driven by"; we agree and have now replaced it with "consistent with".

    Finally, we have rephrased the sentence on evolution at ecological time scales to "especially in microbes, where rapid evolution can occur at short time scales".

11. Line 17 - I don't think this is well-established, although I agree some folks would like it to be. See (Darveau et al. 2002).

    What we meant here was that the Boltzmann-Arrhenius equation is classically treated as mechanistic by the MTE. We have now rephrased this sentence (line 17) to avoid confusion.

12. Line 45 - I think you are overstating the restrictiveness of the UTD. Even Gillooly's original paper (Gillooly et al. 2001) stated a range of eV as "0.2 and 1.2 eV".

    Gillooly et al. 2001 [13] indeed suggest that activation energies should fall between 0.2 and 1.2 eV. However, in later publications in which the MTE framework was more thoroughly described (e.g., Brown et al. 2004 [14] and Gillooly et al. 2006 [15]), the activation energy range was further narrowed to 0.6-0.7 eV. For example, the following sentence is taken from Gillooly et al. 2006:

    > *Second, it predicts that E takes on a limited range of values, 0.6-0.7 eV with an average of about 0.65 eV, reflecting an average activation energy of the biochemical reactions of respiration (Gillooly et al. 2001).*

13. Line 46 - I don't think we really expect a Gaussian distribution for a measurement that cannot be negative, nor is 'very substantial' measurement error needed to generate that right skew. From my experience, even bootstrapped estimates of positive parameters are generally right-skewed without bunching up at 0. I don't think this takes away from the value of your work, but the argument seems to be a bit of a straw man. See also line 354. Maybe it's a matter of degrees.

    We agree, and have rephrased our argument (lines 48–53) to be more along the lines of an argument originally made by Dell et al (2011) [16]:

    > *In principle, right skewness for the rises can be produced by a random diffusion process constrained by a reflective boundary at 0 eV [. . .] The right skewness we observe therefore represents a real and unexplained biological signal.*

14. Line 63 - Seems like you need some references here. How about (Alexander Jr and McMahon 2004).

While Alexander Jr and McMahon 2004 highlight rapid changes in the shape of the TPC of oxygen consumption rate, such changes are the outcome of acclimation to three different temperatures and not adaptive evolution. Nevertheless, we now cite some other relevant studies in line 67.

15. Line 72 - Why does this require 'frequent convergence'? Seems like quickly adapting would be sufficient to break down the ability to detect heritability.

    Rapid adaptive evolution does not necessarily lead to a lack of phylogenetic heritability. For example, major clades could be extremely separated in the parameter space of thermal sensitivity, to the point that adaptive evolution cannot cross the boundaries among them. In that case, the phylogenetic heritability would be significant.

    In contrast, if clades overlap perfectly in thermal sensitivity due to very frequent episodes of convergent evolution, closely related species would not be more similar in thermal sensitivity than randomly chosen species. In such a case, the phylogenetic heritability would be zero. We now clarify this in lines 79–81.

16. Line 141 - If you are doing this analysis phylogenetically, why are you separating the datasets at all? Can't you just run it all together?

    We could indeed merge the phytoplankton and prokaryotic r_{max} datasets and perform the analysis once. However, by analysing them separately, we aimed to examine if the evolutionary patterns of thermal sensitivity differ between planktonic photosynthetic autotrophs and other (autotroph and heterotroph) microbes. We now make this more explicit in lines 177–178.

17. Lines 165-168 - As I understand this here, as well as described by (Harmon et al. 2003), relative disparity varies from 0 to 1, as the subclade disparity is divided by the across-clade disparity. As described by Harmon et al, "Values near 0 imply that subclades contain relatively little of the variation present within the taxon as a whole and that, consequently, most variation is partitioned as among-subclade differences; conversely, values near 1 imply that subclades contain a substantial proportion of the total variation and thus are likely to overlap extensively". This seems to be in line with your description, but my question is that Figure 3 shows a lot of disparity above 1. Can you clarify why this can happen or confirm that there is not an error, or, clarify what I might be missing?

    The disparity-through-time method compares i) the mean disparity within subclades with ii) the total disparity across the phylogeny. Therefore, it is mathematically possible to get values much greater than 1 when the trait variance within subclades (at a given time point) is much greater than the trait variance across the tree. This indeed indicates that clades overlap extensively, to the point that their trait distributions (shown in Fig. 4) are nearly indistinguishable. We now explain this in lines 209–212.

    To avoid confusion, it is also worth stressing that Harmon et al. 2003 analysed 4 clades of lizards and applied this analysis **separately to each clade**. Therefore, in their case, the across-clade disparity stands for the disparity across the entire subtree (subset of the phylogeny) that they analysed each time.

18. Line 172 - 'Exact' might be a stretch, given that the phylogenies and nodes themselves are hypotheses and estimates.

    We have now removed this word.

19. Line 176 - A bit counterintuitive here. Why is adaptive radiation characterized by decelerating evolution? Seems to me like examples of adaptive radiation are generally charac-

terized by fast evolution accompanied by key innovations, which may or may not be part of the disparity. Why not just delete phrase after 'space'?

Adaptive radiations are generally characterised by a brief initial period of rapid trait evolution, followed by a decrease in evolutionary rate with time, due to the filling of ecological niches [17, 18]. We have now rephrased the sentence (lines 217–221) to make this clearer.

20. Line 211 - What does subtree refer to here?

    To perform phylogenetic analyses on each TPC dataset, we first removed any species from the phylogeny for which we did not have TPC parameter estimates. This process is called "pruning", whereas the resulting tree (which is a subset of the entire phylogeny) is called "subtree". We now briefly explain this term when it first appears in the manuscript (in the caption of Fig. 3).

21. Line 214 - I think optimum is the wrong word for the UTD of 0.65. As indicated in the same sentence, the value is thermodynamically-constrained, which is a biochemical/physical property and may or may not be an optimum. Likewise in line 218 and later, I would suggest a 'global mean set by thermodynamic constraints' not a 'global optimum'.

    We agree that the term "optimum" may be misleading in this context, despite it being very often used in the phylogenetic literature to describe models similar to ours. We now use the terms "key central value/tendency" instead.

22. Line 261 - Might want to specify that it's a thorough analysis in prokaryotes and phytoplankton, since otherwise it suggests you have all kinds of species.

    Addressed in line 327.

23. Line 267 - 'are' not 'is' here, I see two subjects.

    We have now rephrased this sentence in lines 332–335.

24. Line 268 - Suggest here adding 'rate-limiting enzyme ... of respiration'.

    Addressed as suggested in lines 332–333.

25. Line 285-286 - Not sure this argument makes sense. E doesn't really tell you breadth on its own. A low E doesn't automatically indicate narrow or wide, because the breadth is determined by the distances between upper and lower critical values and Tpk that could be connected by steep or shallow rises. I would also suggest that Wop is an arbitrary indicator of width making no assumptions about realized environmental temperatures. You might be best off getting rid of this statement, or at least getting clearer on the message.

    The measure of niche width that we use in this study (the "operational niche width"; W_{op}) assumes that species tend to experience temperatures slightly below their temperature of maximum performance. Previous studies suggest that this is a reasonable assumption to make [10, 11], especially given that we do not have field temperature data for any species in our dataset. We now better explain the assumption underlying W_{op} in the caption of Fig. 1.

    W_{op} stands for the difference between T_{pk} and the temperature at the rise of the TPC where $B(T) = \frac{B_{pk}}{2}$. Thus, an increase in W_{op} (a larger operational niche width) will necessarily be associated with a shallower rising slope (a low E value). This is also shown in Fig. S4.

26. Line 310 - Tpk in respiration, the purported underlying driver of rmax, is negatively associated with latitude (DeLong et al. 2018), which I think complicates this explanation a bit, since latitude is invoked as a proxy for fluctuations.

    Both thermal fluctuations and their central tendency (e.g., mean temperature) vary with latitude. Many studies have indeed reported a decrease of T_{pk} with absolute latitude, most likely being the outcome of adaptation to the mean temperature of the environment. In contrast, a systematic decrease of E from low to intermediate latitudes (but not high) is consistent with an increase in the magnitude of thermal fluctuations, which should select for thermal generalists (with low E). We have now rephrased the sentence (lines 390–392) to make this clearer.

27. Line 320 - See (Uiterwaal et al. n.d.) for a recent example of a link between TPC evolution and biotic interactions as well as (Luhring and DeLong 2016) for direct evidence that biotic interactions alter rmax TPCs.

    In this study, we focused on the evolution of thermal sensitivity of lab acclimated/evolved TPCs. Contrary to instantaneous TPCs which reflect the plasticity of a biological trait after brief exposure to different temperatures, our acclimated TPCs were obtained after sufficient acclimation time to each temperature (see also comment #9 and lines 137–139 in the manuscript). Thus, when we referred to biotic interactions as a driver of E shifts, we meant that e.g., predation pressure could lead to non-plastic changes in thermal sensitivity, achieved through novel genetic mutations. Such mutations typically emerge after a high number of generations. For example, a 300-generation selection experiment with *Thalassiosira pseudonana* resulted in 9 likely novel genetic mutations, none of which had an impact on amino acid sequence [19].

    In contrast, the two studies suggested by the Reviewer identified systematic changes in TPC shape after exposure to predation pressure for much fewer than a hundred generations. Given this short amount of time, we do not expect that such TPC variation arose from novel mutations but is a plastic (acclimatory) response. Indeed, the duration of exposure to a new environmental (in the broad sense) factor has been shown to strongly affect the shape of TPCs [20] but such effects are outside the scope of our present study. To avoid confusion, we now use the term "adaptive shifts" in line 417.

28. Line 348 - Not sure I see the mechanistic explanation. Even the previous paragraph explained a range of mechanisms that could underlie the changes in TPCs. Seems to me you've demonstrated patterns of evolution that clearly contradict the UTD but haven't explained the mechanisms.

    In this study, we thoroughly describe the evolutionary patterns of thermal sensitivity. In so doing, we find that rapid evolution driven partly by environmental adaptation mechanistically explains the skewness of the E distribution across species. We note that this is in stark contrast to the widely held assumption of E undergoing neutral (non-adaptive) evolution under strong thermodynamic constraints.

29. Line 358-362 - I don't get this. I don't think the disparity analysis is exactly evidence of convergence, which would require demonstrating that different lineages evolved the same trait in the same conditions. It says that the clades strongly overlap in state space, which may or may not contain cases of convergence.

    There are multiple definitions for the term "convergent evolution" in the literature. A recent study [21] classified them into two major groups:

    > [...] *pattern-based or process-neutral definitions where convergence occurs when lineages independently evolve similar phenotypes, and process-based or causally committed defi-*

nitions where convergence occurs when lineages independently evolve similar phenotypes due to a certain cause or by a certain evolutionary process.

In our case, we use the term "convergent evolution" to refer to remote clades that increasingly overlap in the parameter space due to independent evolution of similar thermal sensitivity values. Our results indeed suggest that this is driven partly by adaptation to similar environmental conditions (Fig. 7). We now make this more explicit in lines 78–79.

Comments by Reviewer #3

30. This study seeks to understand how thermal sensitivity of three key traits among prokaryotes, phytoplankton, and plants evolve along the branches of their respective phylogenetic trees. This is potentially important for knowing how species will respond to human-mediated climate change. The manuscript reports that thermal sensitivity does not evolve in a gradual manner (i.e. via random walk) but can differ considerably even between closely related species, and that there is a weak negative association between thermal sensitivity and latitude.

    The paper is very nicely written - its introduction was a pleasure to read. However, in my opinion, the analyses and methods leave a great deal to be desired. The analyses are poorly described and highly unconventional (which is not a bad thing but in this case seems totally unnecessary and confused). It is not clear at all how the analyses relate to the hypotheses under investigation nor is it clear how various comparative tests should be interpreted and reconciled with each other.

    I think there are some basic problems with the way the authors describe their null model - they often talk about traits evolving randomly; however, it is more accurate to say by random walk or Brownian motion. And even if a trait is evolving by random walk along the branches it does not rule out that the trait has evolved under selection - though this is a relatively minor problem. What is more serious is that the set of three analyses basically describe the same phenomenological pattern in the data but have distinct interpretations - thus the reader is left wondering how to interpret the results.

    We thank the Reviewer for their kind words and for the very constructive comments. We now explicitly describe the hypotheses that we test in this study in lines 94–127. We hope that, in so doing, it is much easier to understand the motivation behind each of our analyses.

    Regarding random evolution, we have now rephrased the main text (e.g., see lines 71–74) to make it more explicit that we refer to a random walk in trait space and not necessarily to the absence of selection. Indeed, a trait evolving similarly to Brownian motion could be tracking a non-stationary optimum that performs a random walk. One such example is T_{pk} (Fig. 2) which arguably responds to the mean temperature that each species experiences in its natural environment [3, 11].

31. The first MCMCGLMM analyses takes all traits together and estimates heritability (h2). It is not clear how the authors constructed these multi-response models to achieve this and the performance of such models has never been assessed. Theoretically they should give identical results to more conventional tests such as Pagel's Lambda - but the prior on the random effects in the MCMCGLMM analyses can be influential. I think it would be better for the authors to conduct a more reliable test using Lambda (with or without estimating covariances among traits - depending on significance given the tree), or at least such a test should be compared with the MCMCGLMM result. In any case if we take the

analyses at face value then it shows that there is some phylogenetic signal in the data, but it is not perfect - i.e. h2 is lower than one. This could be an honest indication that there is lack of signal in the trait or it could be noise in the data. The authors attempt to show that the signal is not owing to noise, but it is not clearly explained and was not convincing to me in its current state.

The full description of the model that we fitted is provided in a different study [3] which we cite at that point in the manuscript. Nevertheless, we do provide a brief description of the model structure in the Methods section, in which we include information on all different model components but priors.

We believe that our approach is far superior to a Pagel's λ analysis for each TPC parameter separately. This is because: i) we explicitly model the likely covariances among all pairs of TPC parameters; ii) we incorporate multiple measurements per species where available, along with their uncertainty; iii) missing values in one response variable can be estimated from other co-varying response variables and the phylogeny (the "Missing At Random" approach), with the uncertainty being incorporated in model coefficients. We are not aware of any other way to estimate Pagel's λ (which is equivalent to phylogenetic heritability [22]) while accounting for these 3 key points. We now mention this explicitly in lines 549–552.

Regarding the influence of priors on the results, Fig. 2 shows that heritability estimates and their 95% HPD intervals vary considerably both among TPC parameters and between the two groups (phytoplankton and prokaryotes). For example, $\ln(E)$ is always less heritable than $\ln(B_{\text{pk}})$, whereas the latter is much more heritable in prokaryotes than in phytoplankton. Another example is that the 95% HPD interval of $\sqrt[4]{B_0}$ is always much narrower than than that of $\ln(E)$. Such results suggest that heritability estimates are not merely dominated by our choice of priors.

Finally, if all variation in E and W_{op} was random noise, then we would not expect to detect any phylogenetic heritability in these parameters. In contrast, the lower bound of the 95% HPD interval was much greater than 0 for all TPC parameters. We now mention this in lines 187–190.

32. They then go on to try to determine if there is rate variation along and among the branches of the trees. To do this they use the 'Free model' and the 'Stable model'. These are strange choices as they don't identify significant shifts in the rate of evolution (whereas several other methods do, see below). The Free model is heavily overparameterized and at best should only be used to provide a rough visualization of rate variation (see Mooers et al, 1999 and Thomas and Freckleton, 2010 - cited in the manuscript). The Stable model was presented to estimate ancestral states rather than rates (although these are two sides of the same coin). Neither method formally identifies areas of the tree (or branches) which are evolving at a significantly different rate, yet this what the authors need to support their conclusion. Methods such as those described in Rabosky (2014, PLoS ONE 9(2): e89543), Landis et al (Systematic Biology 62(2): 193-204), Venditti et al (2011, Nature 479(7373): 393-396), Eastman et al (2011, Evolution 65(12): 3578-3589) can be used to achieve this - this list is not exhaustive . Looking at the results of the 'Free model' and the 'Stable model', most of the rate variation is at the tips of the tree which is what one would expect give the lack of phylogenetic signal in their previous analyses. It does not in itself support the idea that there are intense episodes of adaptation with closely related species having very distinct rates, which is their main conclusion.

Indeed, our motivation for fitting the free and the stable models was to get a broad understanding of variation in the evolutionary rate of thermal sensitivity across the phylogeny.

For example, it is possible that certain clades exhibit consistently higher evolutionary rates than others, and are thus better able to explore the parameter space of thermal sensitivity (lines 118–123).

We followed the Reviewer's advice and have also now fitted a Lévy process model using the "levolution" program [23]. The results are qualitatively identical to those of the free and stable models, as rate shifts with a high enough posterior probability could be detected throughout the entire phylogeny. We have added a figure with the results of this new analysis in Appendix 1 (Fig. S6).

33. The authors' third analyses seem superfluous. Here they estimate a model with an evolutionary optimum - this will again result in a pattern that one would expect if there was a lack of phylogenetic signal in the data. It is not clear what these analyses add to the paper (other than attractive figures) or how they should be interpreted.

    There is no attempt to discriminate between the results of these first three sets of analyses which have very different interpretations despite similarities in patterns. I think the authors need to take a step back and think about how the statistical test they are carrying out relates to the hypotheses. I believe their question can be addressed by a single set of analyses to identify significant shifts in the rate of evolution on the tree using the methods I mentioned previously.

    We now explicitly describe the hypotheses that we test in this study in lines 94–127 and also link each analysis to them (e.g., see lines 267–272), which should now make clear the necessity of these analyses.

    Specifically, we performed these analyses to understand if, despite some isolated episodes of rapid evolution, E and W_{op} values tend to evolve closely around a key central value, as expected by the MTE. More precisely, the central tendency of E should be ≈ 0.32 eV for photosynthetic organisms, and around 0.6-0.7 eV for others. However, Fig. 6 shows that lineages cover a far wider area of parameter space, without being strongly attracted to a central value ($\widehat{\theta}$). It is also worth noting that $\widehat{\theta}$ is much higher than the MTE expectation in every case.

34. The last set of analyses regarding the latitude is also very confusing and unnecessarily complex. There is no need to estimate models with and without the variance-covariance matrix as a random effect. If there is no signal in the data, h2 will be very low - thus the variance explained by the random effect will be negligible and will reduce to be equivalent to the non-phylogenetic test.

    As shown in Fig. 2, the mean phylogenetic heritability estimates of $\ln(E)$ and $\ln(W_{op})$ are slightly above 0.5, and thus not negligible. Given this, we believe that it is more appropriate to fit both phylogenetic and non-phylogenetic variants of the models, and identify the best model according to DIC. It is also worth noting that the best non-phylogenetic model for $\ln(E)$ strongly outperforms its best phylogenetic equivalent in terms of DIC (by more than 45 units), as shown in Table S1.

References

[1] Gilchrist GW. Specialists and generalists in changing environments. I. Fitness landscapes of thermal sensitivity. Am Nat. 1995;146(2):252–270.

[2] Bernhardt JR, Sunday JM, Thompson PL, O'Connor MI. Nonlinear averaging of thermal experience predicts population growth rates in a thermally variable environment. Proc Biol Sci. 2018;285(1886):20181076.

[3] Kontopoulos DG, van Sebille E, Lange M, Yvon-Durocher G, Barraclough TG, Pawar S. Phytoplankton thermal responses adapt in the absence of hard thermodynamic constraints. Evolution. 2020; in press.

[4] Vasseur DA, Yodzis P. The color of environmental noise. Ecology. 2004;85(4):1146–1152.

[5] Pawar SS. Geographical variation in the rate of evolution: effect of available energy or fluctuating environment? Evolution. 2005;59(1):234–237.

[6] Yvon-Durocher G, Allen AP, Bastviken D, Conrad R, Gudasz C, St-Pierre A, et al. Methane fluxes show consistent temperature dependence across microbial to ecosystem scales. Nature. 2014;507(7493):488–491.

[7] Michaletz ST. Evaluating the kinetic basis of plant growth from organs to ecosystems. New Phytol. 2018;219(1):37–44.

[8] Smith TP, Thomas TJH, García-Carreras B, Sal S, Yvon-Durocher G, Bell T, et al. Community-level respiration of prokaryotic microbes may rise with global warming. Nat Commun. 2019;10(1):1–11.

[9] Padfield D, Yvon-Durocher G, Buckling A, Jennings S, Yvon-Durocher G. Rapid evolution of metabolic traits explains thermal adaptation in phytoplankton. Ecol Lett. 2016;19(2):133–142.

[10] Martin TL, Huey RB. Why "suboptimal" is optimal: Jensen's inequality and ectotherm thermal preferences. Am Nat. 2008;171(3):E102–E118.

[11] Thomas MK, Kremer CT, Klausmeier CA, Litchman E. A global pattern of thermal adaptation in marine phytoplankton. Science. 2012;338(6110):1085–1088.

[12] Pawar S, Dell AI, Savage VM, Knies JL. Real versus artificial variation in the thermal sensitivity of biological traits. Am Nat. 2016;187(2):E41–E52.

[13] Gillooly JF, Brown JH, West GB, Savage VM, Charnov EL. Effects of size and temperature on metabolic rate. Science. 2001;293(5538):2248–2251.

[14] Brown JH, Gillooly JF, Allen AP, Savage VM, West GB. Toward a metabolic theory of ecology. Ecology. 2004;85(7):1771–1789.

[15] Gillooly J, Allen A, Savage V, Charnov E, West G, Brown J. Response to Clarke and Fraser: effects of temperature on metabolic rate. Funct Ecol. 2006;20(2):400–404.

[16] Dell AI, Pawar S, Savage VM. Systematic variation in the temperature dependence of physiological and ecological traits. Proc Natl Acad Sci U S A. 2011;108(26):10591–10596.

[17] Schluter D. The ecology of adaptive radiation. OUP Oxford; 2000.

[18] Moen D, Morlon H. From dinosaurs to modern bird diversity: extending the time scale of adaptive radiation. PLoS Biol. 2014;12(5).

[19] Schaum CE, Buckling A, Smirnoff N, Studholme DJ, Yvon-Durocher G. Environmental fluctuations accelerate molecular evolution of thermal tolerance in a marine diatom. Nat Commun. 2018;9:1719.

[20] Rohr JR, Civitello DJ, Cohen JM, Roznik EA, Sinervo B, Dell AI. The complex drivers of thermal acclimation and breadth in ectotherms. Ecol Lett. 2018;21(9):1425–1439.

[21] Stayton CT. The definition, recognition, and interpretation of convergent evolution, and two new measures for quantifying and assessing the significance of convergence. Evolution. 2015;69(8):2140–2153.

[22] Hadfield J, Nakagawa S. General quantitative genetic methods for comparative biology: phylogenies, taxonomies and multi-trait models for continuous and categorical characters. J Evol Biol. 2010;23(3):494–508.

[23] Duchen P, Leuenberger C, Szilágyi SM, Harmon L, Eastman J, Schweizer M, et al. Inference of evolutionary jumps in large phylogenies using Lévy processes. Syst Biol. 2017;66(6):950–963.
