# Supplementary material for: Adaptive evolution shapes the present-day distribution of the thermal sensitivity of population growth rate
Source: PLoS Biol. 2020 Oct 16;18(10):e3000894. doi: 10.1371/journal.pbio.3000894 (PMC7592915; doi:10.1371/journal.pbio.3000894)
Supplement: Attachment — Submitted filename: responses_to_the_Reviewers.pdf [file pbio.3000894.s004.pdf]

Responses to the Reviewers

Comments made by Reviewer #2 and #3 are in **dark orange** and **purple** respectively whereas our responses are in **black**.

Comments by Reviewer #2

1. The disparity levels going above 1 still seems confusing. The authors write "The disparity-through-time method compares i) the mean disparity within subclades with ii) the total disparity across the phylogeny. Therefore, it is mathematically possible to get values much greater than 1 when the trait variance within subclades (at a given time point) is much greater than the trait variance across the tree." What I don't understand is how there can be more variance within than across subclades, when the variation across subclades would seem to me to be inclusive of all the variation occurring within subclades. I suspect there is some crucial detail missing.

   The Reviewer refers to our response to one of their previous comments and not to the manuscript itself. We agree that our response was ambiguous due to an omission of the word "mean" when we referred to the variance of trait values within subclades. The sentence in our previous response would be better rephrased as:

   "... it is mathematically possible to get values much greater than 1 when the *mean of the trait variances* within subclades (at a given time point) is much greater than the variance of trait values across the entire tree."

   For example, consider two subclades **A** and **B**, with trait values $\mathbf{A} = (2, 6, 15)$ and $\mathbf{B} = (2, 4, 20)$. The variances of **A** and **B** are 44.33 and 97.33 respectively with an average (i.e., the mean subclade variance) of 70.83. At the same time, the variance across all trait values is 56.97 and thus lower than the mean subclade variance.

   We have now also rephrased the main text of the manuscript (lines 218–221) to make this point even clearer.

2. The use of Wop and the link between E and breadth is still confusing. There are two issues.

   A) First, choosing Wop as you have done does not require an assumption. It is just a choice. I think what you might be trying to say is that you chose this specific metric (instead of, say, the difference between the upper and lower critical temperatures) because you feel this metric best captures the curve's breadth in the typical range of experienced temperatures.

   We agree with the Reviewer on point A and have now rephrased the caption of Fig. 1 to better clarify why we chose W_{op} for this study instead of the full thermal niche width.

   B) Second, the explanation that E reflects breadth still doesn't make sense to me. The authors write "an increase in Wop (a larger operational niche width) will necessarily be associated with a shallower rising slope (a low E value)." Yet it is easy to show that this is not true. Consider the attached cartoon.

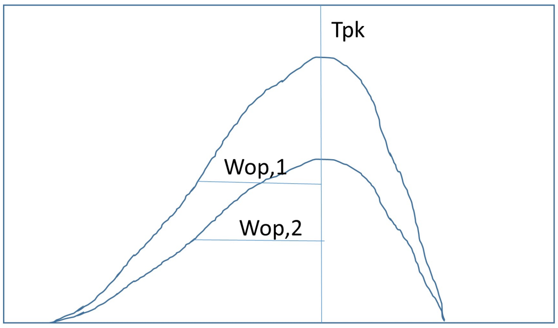

Two curves, same Tpk. The lower curve has a lower E and a smaller Wop - opposite of the 'necessary' pattern. So I find this confusing, and it influences interpretation of the results as well. For example, the explanation for Figure S4 is that it has to be that way. Does it? I think, perhaps, the authors are making additional unstated assumptions about correlations among parameters, such that the proposed necessary correlation will arise under particular scenarios, such as, for example, Tpk and E are negatively correlated.

In general, the example given by the Reviewer is possible if TPCs are prescribed by any of a number of arbitrary unimodal mathematical functions (e.g., a cubic polynomial). However, it is not possible for TPCs prescribed by the Sharpe-Schoolfield model, subject to certain constraints which make the model appropriate for empirical TPCs. Specifically, in the Sharpe-Schoolfield model, W_{op} *necessarily* decreases with E, provided that other parameters (B_0, T_{pk}, and E_{D}) do not systematically and strongly change with E. This constraint indeed exists empirically: in experimentally-determined TPCs, the fact that $E < E_{\text{D}}$, and the existence of correlations between certain TPC parameters (which we have previously established [1]) prevent B_0, T_{pk}, and E_{D} from changing systematically and strongly with E. The resulting expected negative relationship between W_{op} and E is illustrated in Figure 1. We now include this Figure in the S1 Appendix to explain why the empirically observed negative relationship between W_{op} and E is expected, and also clarify this issue in lines 375–379 of the main text.

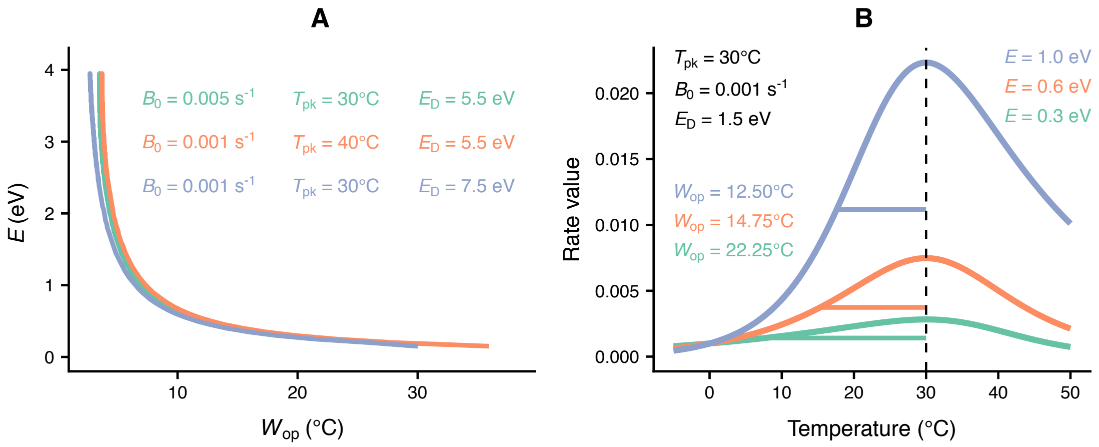

Figure 1: The expected relationship of the operational niche width (W_{op}) and E in the Sharpe-Schoolfield model. A: W_{op} always decreases with E, provided that the other parameters (B_0, T_{pk}, and E_{D}) do not vary substantially and systematically with E. This is illustrated here with three arbitrary fixed combinations of the other three parameters; the curves remain practically the same, irrespective of substantial variation in these other parameters. B: An example illustrating how W_{op} always decreases with E when the other parameters are fixed (values shown in black). As E increases from 0.3 to 1 eV, W_{op} decreases from 22.25 to 12.50°C.

3. It is still not very clear why the disparity analysis indicates convergent evolution. The authors clarify that there are different definitions of convergence that focus on pattern or process, and that they use the term "convergent evolution" to refer to remote clades that increasingly overlap in the parameter space due to independent evolution of similar thermal sensitivity values. Note the point here is 'similar thermal sensitivity values'. To me the disparity results seem to say that the subclades have evolved similar 'ranges' - that is, a lot of overlap in extent - rather than showing convergence toward similar values within that extent. The pattern appears to be (and this is a major point of the paper) that lineages are exploring a lot of thermal parameter space. Overlapping a lot because multiple lineages have evolved a wide range of parameter values does not seem to me to be consistent with the idea of convergent evolution.

   The thermal sensitivity distributions of evolutionarily remote phyla (e.g., Bacillariophyta and Proteobacteria; Fig. 4 in the main text) are not only similar in their ranges but also in their shapes, i.e., the same values are found at similar frequencies across the distributions, and their central tendencies are similar. This indicates convergence, which we hypothesize is due to selection for similar thermal sensitivity strategies. We now make this clearer in lines 239–241.

4. Figure 1 legend - "E and ED (eV) control how smoothly". Isn't it how steeply, since a low E does not cause a 'rough' rise?

   We have now changed "smoothly" to "steeply".

5. L123 - Evolution would not favor species but individuals with lower thermal sensitivities.

   Addressed.

6. L214 - The 'true' disparity is not really knowable. The 'estimated' or 'observed' disparity is - 'observed' is the word you used in the F3 legend. I would use that.

   Addressed.

Comments by Reviewer #3

7. The authors are misguided in their assessment: the MCMCglmm analyses are not far superior. h2 and Lambda are mathematically identical (if for lambda you take a mean of the within-species values and estimate co-variances among traits) - this is clear in the original paper describing heritability in the context of phylogenetic analysis, which the authors cite for a description of the model! My specific worry is that in the MCMCglmm implementation, h2 can be very sensitive to the prior. (Incidentally, what prior was on the random effect here, and what does the distribution look like if you turn the likelihood function off and just target the prior?) This sensitivity to prior choice is not the case for Lambda, owing to the fact that it is implemented within the more conventional GLS framework. I therefore still maintain that given this, it is important to check the test using the more well-established method. Estimating co-variances and Lambda can be done in many GLS packages including BayesTraits - Pagel's own program. Incidentally, phylogenetic imputation can also be done in a GLS framework again in number of packages including BayesTraits.

   What we meant was that simultaneously estimating the phylogenetic heritabilities of multiple traits that covary should lead to more accurate results compared to those obtained by estimating the phylogenetic heritability of each trait in isolation. This is because our TPC parameter dataset contains a number of missing values. Their estimation should be

more accurate if the covariances among TPC parameters are taken into account. We now clarify this in lines 569–573.

For the random effect and the residual variance components of our MCMCglmm fits, we used an inverse Γ prior with a shape and scale of 0.001. This is described in [1] which we cite in line 553.

Following the Reviewer's advice, we also estimated λ in a non-Bayesian way. For this, we did not use BayesTraits but the R package Rphylopars (v. 0.2.12) [2]. This is because BayesTraits can accommodate multiple measurements per species only when MCMC is used. Instead, Rphylopars can include multiple measurements (but not the uncertainty of each measurement) and can phylogenetically impute missing values. However, to obtain a λ estimate for each TPC parameter, each parameter needs to be analysed separately. As a result, the covariance among TPC parameters cannot be taken into account during the estimation of missing values. In any case, the λ values that we obtained with Rphylopars were all greater than 0 for every single TPC parameter across phytoplankton and prokaryotes. In particular, the lowest λ value was for $\ln(W_{\text{op}})$ of phytoplankton and was estimated at 0.12. These results indicate that it is indeed appropriate to use phylogenetic comparative analyses to analyse the distribution of $\ln(E)$ and $\ln(W_{\text{op}})$ across species. Finally, differences in λ values between MCMCglmm and Rphylopars (Fig. 2) do not alter the conclusions of our study, given that both methods agree that thermal sensitivity measures (E and W_{op}) are phylogenetically heritable, but less than T_{pk}. We have now added this extra analysis to the manuscript (lines 182–186, 206–209, and 567–573) and its S1 Appendix.

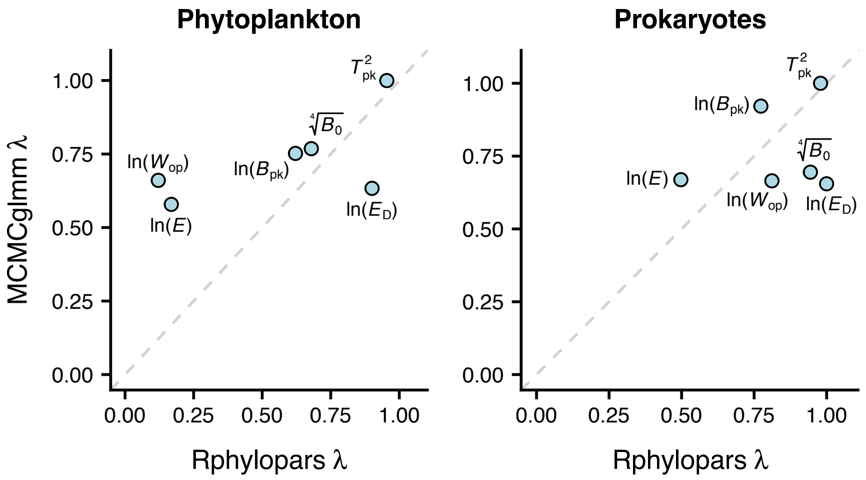

Figure 2: Phylogenetic heritability estimates of TPC parameters, obtained with MCMCglmm and Rphylopars. The dashed line is the one-to-one line. In contrast to Rphylopars, MCMCglmm takes into account the uncertainty of each TPC parameter estimate and the covariances among TPC parameters. TPC parameters that have strong phylogenetic heritability according to MCMCglmm (e.g., T^2_{pk}) also have a high Rphylopars λ estimate. The λ estimates of the two methods mainly differ for parameters that are less phylogenetically heritable and for which many values are missing (e.g., $\ln(W_{\text{op}})$ in phytoplankton).

8. Given that the Levy process analyses have been conducted and are by far better justified I believe they should be reported in the main text - the others can be removed altogether as they are no longer needed.

We think that the Lévy figure (where each branch is coloured according to the posterior

probability for a jump in thermal sensitivity; Fig. S9) is much harder for a general reader to interpret compared to the distribution of evolutionary rates estimated using the stable model (Fig. 5 in the main text). In any case, both analyses lead to the same qualitative conclusion, i.e., that rapid changes in thermal sensitivity occur sporadically across various clades. For these reasons, in the main text we describe the results of all three models (free, stable, and Lévy; lines 265–270) and show a figure with the estimates of the stable model. Figures from the free and Lévy analyses are available in the S1 Appendix.

9. I still can't see the value of these analyses if authors believe the previous analyses - they all show the same thing - but the interpretations are not consistent. I believe they must be removed - how can these results and the Levy process results be reconciled?

   The Lévy process model showed that rapid changes in thermal sensitivity occur sporadically throughout the phylogeny. On its own, this result does not tell us if i) the central tendency of thermal sensitivity values is similar to the MTE expectation and if ii) the jumps in thermal sensitivity are large enough to often lead to values significantly far from the central tendency. It is for these reasons that we performed the analysis shown in Fig. 6 in the main text. We mention these in lines 278–279, which we have now rephrased for clarity.

10. The reasons I state above mean we cannot believe the h2 until it has been verified by lambda so this point still stands.

    See comment #7 above. As we show there, the λ values of $\ln(E)$ and $\ln(W_{\text{op}})$ that we estimated with Rphylopars were greater than zero for both phytoplankton and prokaryotes.

References

[1] Kontopoulos DG, van Sebille E, Lange M, Yvon-Durocher G, Barraclough TG, Pawar S. Phytoplankton thermal responses adapt in the absence of hard thermodynamic constraints. Evolution. 2020;74(4):775–790.

[2] Goolsby EW, Bruggeman J, Ane C. Rphylopars: Phylogenetic Comparative Tools for Missing Data and Within-Species Variation; 2019.
