# Supplementary material for: Adaptive evolution shapes the present-day distribution of the thermal sensitivity of population growth rate
Source: PLoS Biol. 2020 Oct 16;18(10):e3000894. doi: 10.1371/journal.pbio.3000894 (PMC7592915; doi:10.1371/journal.pbio.3000894)
Supplement: Attachment — Submitted filename: response_to_the_reviewer.pdf [file pbio.3000894.s005.pdf]

Response to the Reviewer

I find the difference between Lambda and H2 quite alarming - and I do not really understand why the authors would not use the MCMC option in a Bayesian context for comparison which allows multiple data points per taxa - especially as MCMCglmm also provides a posterior distribution of h2. It would be ideal to see the two distributions together. Such differences in the level of phylogenetic signal have the potential to change the results altogether - I am not sure if that is the case here as I don't have enough information. I think this is a critical point as phylogenetic signal is fundamental to this study. I feel like I am sounding like a broken record here, but the analyses I am suggesting a very easy to do and I can't understand why the authors don't want to perform/show them.

I very much like this paper and hope to see it published on PLoS Biology - but I think this final piece of the puzzle is missing.

We thank the Reviewer for their kind comments and for encouraging us to put the final piece of the puzzle in place. Before we do so, we would like to first clarify a misunderstanding. The only scenarios in which the phylogenetic heritability estimates would alter the conclusions of the present study are the following:

- A complete absence of phylogenetic heritability in $\ln(E)$ and $\ln(W_{\text{op}})$ for both phytoplankton and prokaryotes (see lines 101–103 and 349–351 in the main text).
- Higher phylogenetic heritability in $\ln(E)$ and $\ln(W_{\text{op}})$ than in T_{pk}^2 (see lines 386–397).

With these in mind, we will now give a brief summary of the approaches that we used to estimate phylogenetic heritability for this manuscript. In our initial submission, we used MCMCglmm (a Bayesian method), as it can account for i) multiple measurements per species, ii) the uncertainty around each measurement, and iii) the likely covariances among pairs of TPC parameters. Using MCMCglmm, missing values in a TPC parameter can be estimated not only based on known values from other species, but also based on the covariance of the TPC parameter with other parameters for which values are available. This analysis showed that all TPC parameters exhibit at least moderate phylogenetic heritability, with that of T_{pk}^2 being the highest. Additionally, most TPC parameters are similarly phylogenetically heritable among phytoplankton and prokaryotes.

The Reviewer suggested that we also estimate phylogenetic heritability in a non-Bayesian way to ensure that our results are not mainly dominated by the priors. For this, we used Rphylopars which can accommodate multiple measurements per species, but not their uncertainty estimates or the covariances among TPC parameters. The phylogenetic heritability estimates obtained with Rphylopars are greater than zero for all TPC parameters, whereas $\ln(E)$ and $\ln(W_{\text{op}})$ are much less phylogenetically heritable than T_{pk}^2. Nevertheless, there is not a perfect correlation between the estimates of MCMCglmm and those of Rphylopars, most likely due to the aforementioned limitations of the latter method.

Following the Reviewer's encouragement, we have now also estimated phylogenetic heritabilities using BayesTraits' Bayesian mode. After contacting Andrew Meade (the lead developer of BayesTraits), we confirmed that BayesTraits cannot account for covariance among TPC parameters or measurement uncertainty, similarly to Rphylopars. The resulting posterior distributions of phylogenetic heritability (Pagel's λ) are shown in the Figure below.

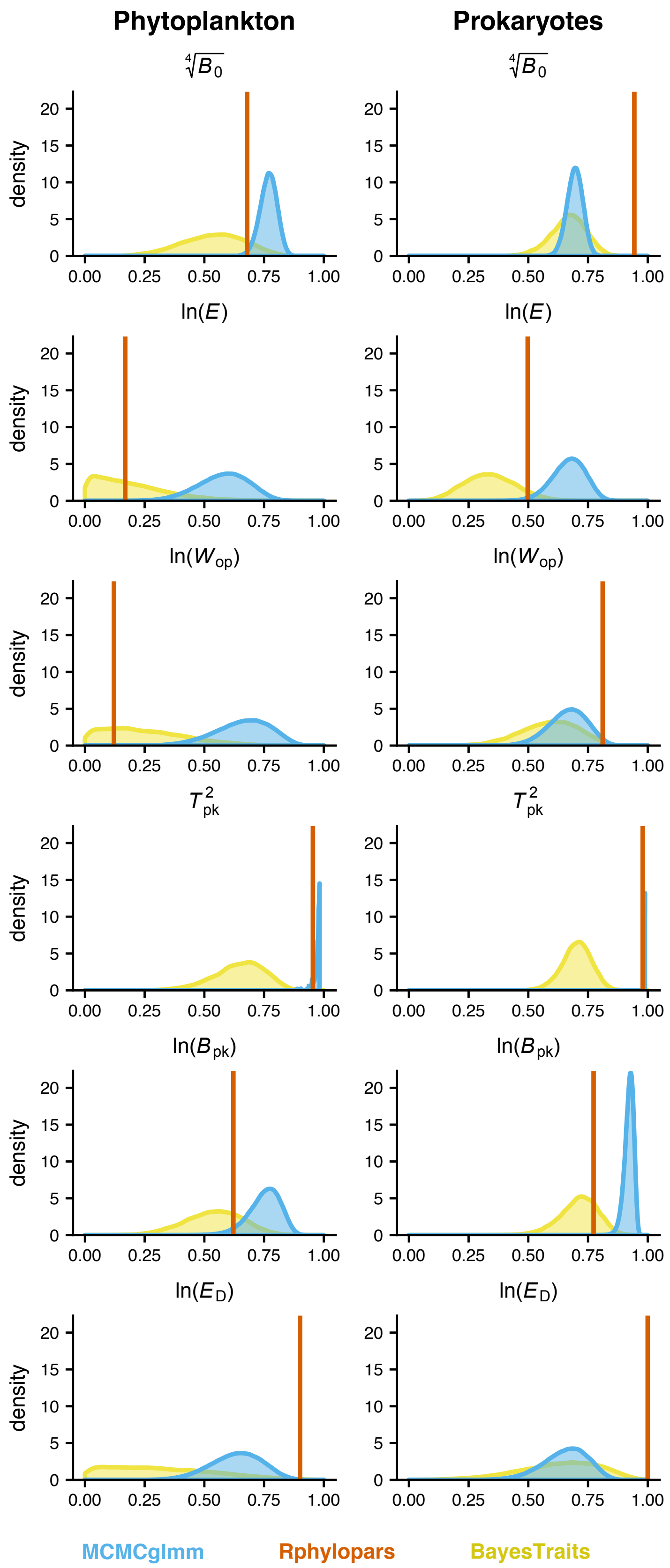

Figure 1: Phylogenetic heritability estimates of TPC parameters, obtained with MCMCglmm, Rphylopars, and BayesTraits. As MCMCglmm and BayesTraits estimate phylogenetic heritability using a Bayesian approach, the plots show their posterior distributions. Instead, point estimates are shown for Rphylopars.

Based on this Figure, a number of conclusions can be extracted. First, despite the similarity in the approach employed by Rphylopars and BayesTraits, their estimates are often strongly different. This disagreement most likely arises from differences in the way that the two programs reconstruct missing trait measurements. Second, the mean phylogenetic heritability estimates of BayesTraits are generally lower than those of MCMCglmm and Rphylopars, albeit the lower bound of the 95% Highest Posterior Density interval of BayesTraits is always greater than zero (the lowest value is at $2 \cdot 10^{-5}$ for $\ln(E)$ among phytoplankton). Third, BayesTraits agrees with the other two programs that T_{pk}^2 is more phylogenetically heritable than $\ln(E)$ and $\ln(W_{\text{op}})$. Fourth, the distributions of phylogenetic heritability estimates of prokaryotes obtained with BayesTraits are much narrower and closer to those of MCMCglmm, compared to those obtained for phytoplankton with the two programs.

All these results in combination strongly highlight the importance of how uncertainty is treated during the estimation of phylogenetic heritability. More precisely, it is striking that MCMCglmm and BayesTraits have more similar posterior distributions for the dataset of prokaryotes than for that of phytoplankton. This result is consistent with an increase in the "signal-to-noise" ratio from phytoplankton to prokaryotes, given that the latter dataset is larger (see Figure S2 in the Appendix). It is also worth stressing that, for most TPC parameters, MCMCglmm yielded similar phylogenetic heritability estimates among phytoplankton and prokaryotes, whereas BayesTraits (and Rphylopars to some extent) generally did not. The improved performance of the MCMCglmm approach is also visible when comparing the posterior distributions of phylogenetic heritability of prokaryotes between MCMCglmm and BayesTraits. Even when their means are very close (e.g., for $\sqrt[4]{B_0}$ or $\ln(E_{\text{D}})$), the distribution of MCMCglmm is much narrower.

Overall, these analyses show that as the size of the dataset increases, the phylogenetic heritability estimates of Rphylopars and BayesTraits approach those of MCMCglmm. This is expected as MCMCglmm additionally accounts for the uncertainty of each measurement and for the correlations among TPC parameters when estimating missing values. We have now added the BayesTraits analysis to the main text (lines 184–186, 206–207, and 565–573) and the Figure to the S1 appendix.
