# Supplementary material for: Adaptive evolution shapes the present-day distribution of the thermal sensitivity of population growth rate
Source: PLoS Biol. 2020 Oct 16;18(10):e3000894. doi: 10.1371/journal.pbio.3000894 (PMC7592915; doi:10.1371/journal.pbio.3000894)
Supplement: Attachment — Submitted filename: response_to_the_reviewer.pdf [file pbio.3000894.s006.pdf]

Response to the Reviewer

One can see that the results of the phylogenetic signal are quite different from different methods! As the authors are happy to include this and discuss them I am happy to recommend publication – with one caveat. The authors seem to think that the difference is associated with the way the program deal with missing data. I doubt very much this is the case – one should not be able to get more information about signal from imputed/missing data points! I am sure that the difference between mcmcglmm and Baystraits is the prior. The prior is far more simple to implement in a GLS framework (BayesTraits) compared to the way it has to be implemented in mcmcglmm. I think the authors should at least posit this idea – then the reader can decide and/or test it in the future.

We now explicitly refer to the priors used by MCMCglmm and BayesTraits and their likely influence on the phylogenetic heritability estimates, in the caption of Fig F in the S1 Appendix. We agree with the Reviewer that this can be investigated further in future studies.
